# Synthesis, characterization, and nonlinear optical properties of copper (II) ligand Schiff base complexes derived from 3-Nitrobenzohydrazide and benzyl

**DOI:** 10.1038/s41598-023-38086-w

**Published:** 2023-07-07

**Authors:** Ayub Tahmasbi, Akbar Jafari, Abbas Nikoo

**Affiliations:** 1 Atomic and Molecular Group, Department of Physics, Faculty of Science, Urmia University, Urmia, Iran; 2 Department of Organic Chemistry, Faculty of Chemistry, Urmia University, Urmia, Iran

**Keywords:** Chemistry, Materials science, Nanoscience and technology, Optics and photonics, Physics

## Abstract

A new series of Cu (II) complexes were prepared using Schiff base ligand of *N*–*N′*-(1,2-diphenyl ethane-1,2-diylidene)bis(3-Nitrobenzohydrazide). The prepared ligand and Cu (II) complex were characterized using various physicochemical investigations such as X-ray diffraction (XRD), Field emission scanning electron microscopy (FESEM), and Energy dispersive X-ray analysis (EDX), Fourier Transform Infrared (FT-IR), ${}^{13}C$ Nuclear Magnetic Resonance (NMR), ${}^{1}H$ NMR, Diffuse Reflectance Spectroscopy (DRS), Vibrating Sample Magnetometer (VSM), and Z-Scan technique (Nonlinear optical (NLO) properties). In addition, the prepared samples have been examined for their NLO characteristics with the help of the Density Functional Theory calculations which proved that the Cu (II) Complex is more polarized than Ligand. According to XRD and FESEM results, the nanocrystalline nature of the samples is confirmed. The metal-oxide bond assigned in the functional studies by FTIR. Magnetic studies demonstrate weak ferromagnetic and paramagnetic nature for Cu (II) complex and diamagnetic nature for the ligand, respectively. DRS spectrum exhibited higher reflectance for Cu (II) than the ligand. The band gap energies of the synthesized samples were estimated by employing the Tauc relation and Kubelka–Munk theory on reflectance data and found to be 2.89 eV and 2.67 eV for Cu (II) complex and ligand, respectively. Extinction coefficient and refractive index values were calculated using the Kramers–Kronig method. The z-scan technique was applied to estimate the NLO properties by a 532 nm Nd:YAG laser.

For the first time, the expression "Schiff bases" was used in 1864, since Hugo Schiff, a Nobel Prize laureate, and scientist, prepared the Schiff base (Sb) through a condensation reaction of carbonyl functionality (ketone or aldehyde) and primary amines^1^. The Schiff bases (Sbs) got great attention recently because of their optical application in nonlinear optics (NLO)^2^, fluorescence^3^, electroluminescence^4^, and biological applications like antibacterial activities^5^. With the help of most transition metals, Sbs could easily create a stable complex^6^. As ligands, Sbs successfully used in coordination chemistry due to the broad chelating potential of most metal ions and their facile preparation^7^. Basicity, strength, and steric of the azomethine group affect the Sb complex's stability^8^**.** Schiff bases are renowned for their diverse catalytic and biological applications, they represent a class of ligands that exhibit a broad spectrum of utility in coordination chemistry^9^. Schiff base derivatives of transition metal complexes have garnered considerable attention as oxidation catalysts for alcohols and alkenes owing to their inexpensive and facile synthesis as well as their remarkable chemical and thermal stability. Schiff bases metal complexes are regarded as a very essential type of organic compounds, which have extensive applications in various biological aspects anti-bacterial, antitumor, antifungal, anti-cancer, anti-tuberculosis, DNA binding, analgesic, antioxidant, and anti-viral properties^10,11^. These tremendous applications of Schiff bases have provided a great deal of interest in Cu(II) complexes. Moreover, copper (II) complexes have been demonstrated to be highly effective catalysts for the oxidation of benzyl alcohol^12^. Copper (II) complexes have been prepared for their potential utilization in diverse medicinal applications, including cytotoxic, antifungal, antibacterial, DNA photocleavage, anticancer, antitumor, and antioxidant activities^13–15^. The optical beam’s frequency, polarization, amplitude, and phase could be affected by NLO materials. Besides, these materials presented a crucial large third or second optical susceptibilities^16^. To provide NLO materials, Schiff base is the most suitable procedure^17^. The NLO plays a crucial role in the recent technological improvements in plasma physics^18^, quantum computing^19^, second harmonic generation^20^, and Q-switching^21^. Besides, NLO materials have applications in sorely fast optical modulation and switching^22^. The most popular method for determining the NLO properties in materials includes Z-scan^23^, I-scan^24^, and two-beam coupling^25^. Compared with other methods, Z-scan was widely used because of its high sensitivity and simplicity^26^. In 1989 Sheikh-Bahaei et al*.* expressed the Z-scan method to study the NLO characteristics of materials^27^. Moreover, this technique, with the help of a beam, can make a single sensitive analysis for both nonlinear refraction and nonlinear absorption at the same time^23^. By applying the Z-scan technique, we can approach the high simplicity and accuracy of third-order susceptibility $\left( {\chi^{(3)} } \right)$, nonlinear absorption $\left( {NLA,\beta } \right)$, and nonlinear refraction $\left( {NLR,n_{2} } \right)$^28^.

On the other hand, among the experimentally determining nonlinear susceptibilities techniques of intensity-dependent materials like organic materials^29^, nanoparticles^30^, metal crystals^31^, and organometals^32^, the Z-scan technique is broadly used. Compared to other NLO Sb materials, Hydrazides were identified as an adjustable and suitable compound^33^. In this regard, Albayati et al*.*^34^ synthesized benzo hydrazide (BH) and characterized it theoretically and experimentally. Also, another BH material was synthesized and characterized structurally by Babu et al*.*^35^. Latha et al*.*^17^ reported optical, spectral, and Structural properties of the hydrazide-based Schiff bases. They used the Z-Scan method to study the third harmonic for optical usages. Ola A. El-Gammal et al*.* prepared and investigated other BH-based Sb ligands and their metal complexes for their antibacterial, DNA binding, and catalytic activities^36^.

According to the literature and to the best of our knowledge, there is not adequate research on using the Z-scan technique to determine nonlinearly properties of the ligand Schiff bases and their complexes. Furthermore, there is not any exclusive research on the structural, functional, ${}^{1}H$ and ${}^{13}C$ NMR, magnetic, nonlinear, and linear optical properties of Cu (II) ligand Schiff base complexes derived from 3-Nitrobenzohydrazide and benzyl. Due to the applications mentioned above, it seemed to be worthwhile to prepare and investigate the *N*–*N′*-(1,2-diphenylethane-1,2-diylidene)bis(3-Nitrobenzohydrazide) and its complex with Cu (II). In this regard, the present study introduces ligand Sbs and its complex with Cu (II) preparation method. The synthesized powders have been examined via X-ray diffraction (XRD), Fourier Transform Infrared (FT-IR), ${}^{13}C$ Nuclear Magnetic Resonance (NMR), ${}^{1}H$ NMR, Diffuse Reflectance Spectroscopy (DRS), Vibrating Sample Magnetometer (VSM), Z-Scan, and Density Functional Theory (DFT) techniques for the first time in the literature.

## Experimental

### Preparation of ligand and its complex

#### Procedure for the synthesis of N–N′-(1,2-diphenylethane-1,2-diylidene)bis(3 nitrobenzohydrazide)

Benzil (1 mmol, 210.2 mg) was dissolved in ethanol (100 mL) with stirring. 3-Nitrobenzohydrazide (2 mmol, 362 mg) was added, and the reaction mixture was heated under reflux for 48 h. TLC eluted with ethanol/toluene. The constituted precipitate was filtered off and washed with ethanol. The resulting solid was dried in the air and recrystallized from DMF/H_2_O. Chemical formula: $C_{28} H_{20} N_{6} O_{6}$, Colour: yellow; Yield: 0.35 g, 65%, m.p.: 143.5–145.5 °C, IR (KBr, cm^−1^): 3274, 3084, 3051, 1686, 1663, 1528, 1451, 1348, 1291, 1275, 1217, 1152, 1086, 1067, 870, 744, 717, 702, 677, 655, 583; ^1^H NMR (400 MHz, DMSO-*d*_*6*_) δ (ppm): 7.54–7.58 (8H, m, ArH), 7.72 (2H, s, ArH), 7.79 (2H, s, ArH), 8.12 (2H, d, *J* = *7.6* = Hz, ArH), 8.36 (2H, d, *J* = 8 Hz, ArH), 8.47 (2H, s, ArH), 11.35 (2H, bs, NH); ^13^C NMR (100 MHz, DMSO-*d*_*6*_) δ (ppm):123.31, 126.00, 128.16, 128.86, 128.92, 129.32, 129.83, 130.03, 130.29, 132.97, 134.87, 136.23, 147.24, 191.63. Elemental. Calcd: C, 62.68; H, 3.76; N, 15.66. Found: C, 62.51; H, 3.81; N, 15.41%.

#### Procedure for the synthesis of complex

The ethanol solvent is cheap, available, and less toxic than other solvents. Ethanol dissolves the ligand at boiling temperature and due to its miscibility with water, it is suitable for the synthesis of the complex. To the solution of *N*–*N′*-(1,2-diphenylethane-1,2-diylidene)bis(3-Nitrobenzohydrazide) (1 mmol, 540 mg) in ethanol (100 mL), CuSO_4_·5H_2_O (1 mmol, 250 mg) dissolved in distilled water (10 mL) was added. The above reaction mixture was stirred and heated under reflux for six hours. After completion of the reaction, the formed precipitate was filtered off and rinsed with ethanol and dried, and after that, recrystallized using ethanol. Chemical formula:

C_28_H_24_CuN_6_O_12_S, Colour: dark green; Yield: 0.61 g, 83%, m.p.: 184–186 °C, IR (KBr, cm^−1^): 3205, 3079, 3057, 1682, 1525, 1348, 1263, 1150, 1067, 754, 716, 689; Elemental. Calcd: C, 45.93; H, 3.30; N, 11.48; S, 4.38. Found: C, 45.52; H, 3.81; N, 11.21; S, 4.11%.

**Figure 1:**
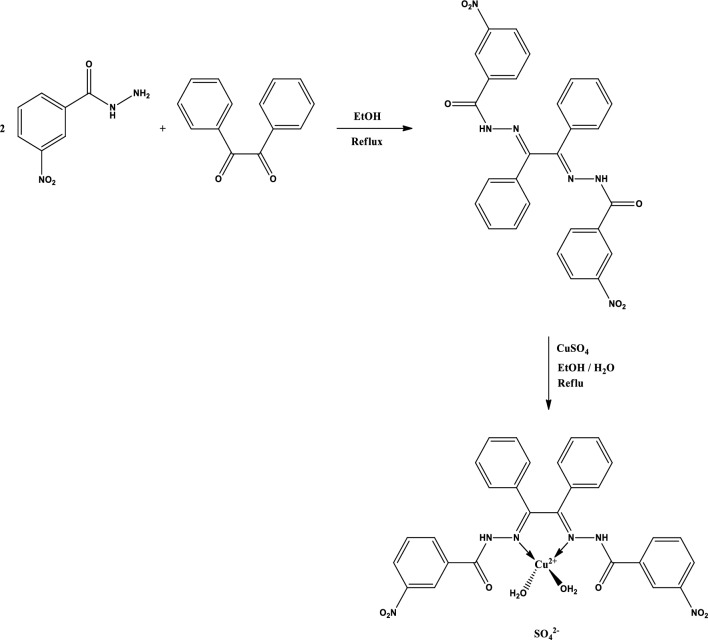
Schematic route for the synthesis of ligand and Cu (II) Complex.

The schematic route for the synthesis of the Cu (II) complex and Schiff base ligand is shown in Fig. 1. The Schiff base and its Cu (II) complex were prepared in good yield (Fig. 1), the physical properties of the synthesized Schiff base and its Cu (II) complex were analyzed and presented in Tables 3 and 4. The percentage yield of the Schiff base was 65% while that of the complex was 83%. The Schiff base was yellow while the Cu (II) complex was dark green, respectively. It was found that the melting point of the Schiff base is 143.5–145.5 °C and the decomposition temperature of the Cu (II) complex is 184–186 °C, this is an indication of thermal stability.

### Apparatus

The chemicals are produced by Sigma-Aldrich and Merck. Before use, the materials are purified by employing standard processes. TLC analysis was performed on precoated silica gel (E-Merck kieselgel 60 F254 Aluminium sheets) plates. With the help of a digital melting point (m.p.) instrument, melting points were ascertained in open capillaries. Fourier Transform Infrared (FT-IR) spectra are recorded KBr on Thermo Nicolet Nexus 670 FT-IR. By utilizing Bruker Avance AQS 300 MHz spectrometer at 100 MHz and 400 MHz, ^13^C Nuclear Magnetic Resonance (NMR), and ${}^{1}H$ NMR spectra were determined, respectively. The polarity of the ligand is such that it dissolves only in DMSO and DMF at room temperature. Measuring DMSO-*d*_*6*_ as a solvent relative to TMS as the internal standard helped to determine the chemical shifts. X-ray diffraction (XRD) provided helpful information about the structural properties of the complex. Hence the structural analysis was carried out through the XRD technique with a Cu Kα source of radiation. To collect information about the morphology, elemental mapping, and the particles size of ligand and Cu (II) complex, FESEM analysis was performed with a Field Emission Scanning Electron Microscope (FESEM, TESCAN MIRA III) with 15 kV accelerating voltage. Energy Dispersive X-ray analysis (EDX) using a SAMX detector was used to understand the distribution of metals over the ligand and Cu (II) complex. Vibrating sample magnetometer (VSM) analysis was carried out at RT in a magnetic field from –15 to + 15 kOe. To evaluate linear and nonlinear optical properties, Diffuse Reflectance Spectroscopy (DRS) and Z-scan techniques were employed. The linear refractive index (n_0_) values were obtained using a diffractometer.

### Computational studies

One of the most significant methods to examine the electronic structure is DFT^37^. All the electronic calculations were performed using Gaussian 16 Software^38^. The ligand and Cu (II) complex are theoretically optimized by B3LYP level along with a 6–311G basis set and the computation on their frequency is carried out.

## Results and discussion

### Structural investigations

**Figure 2:**
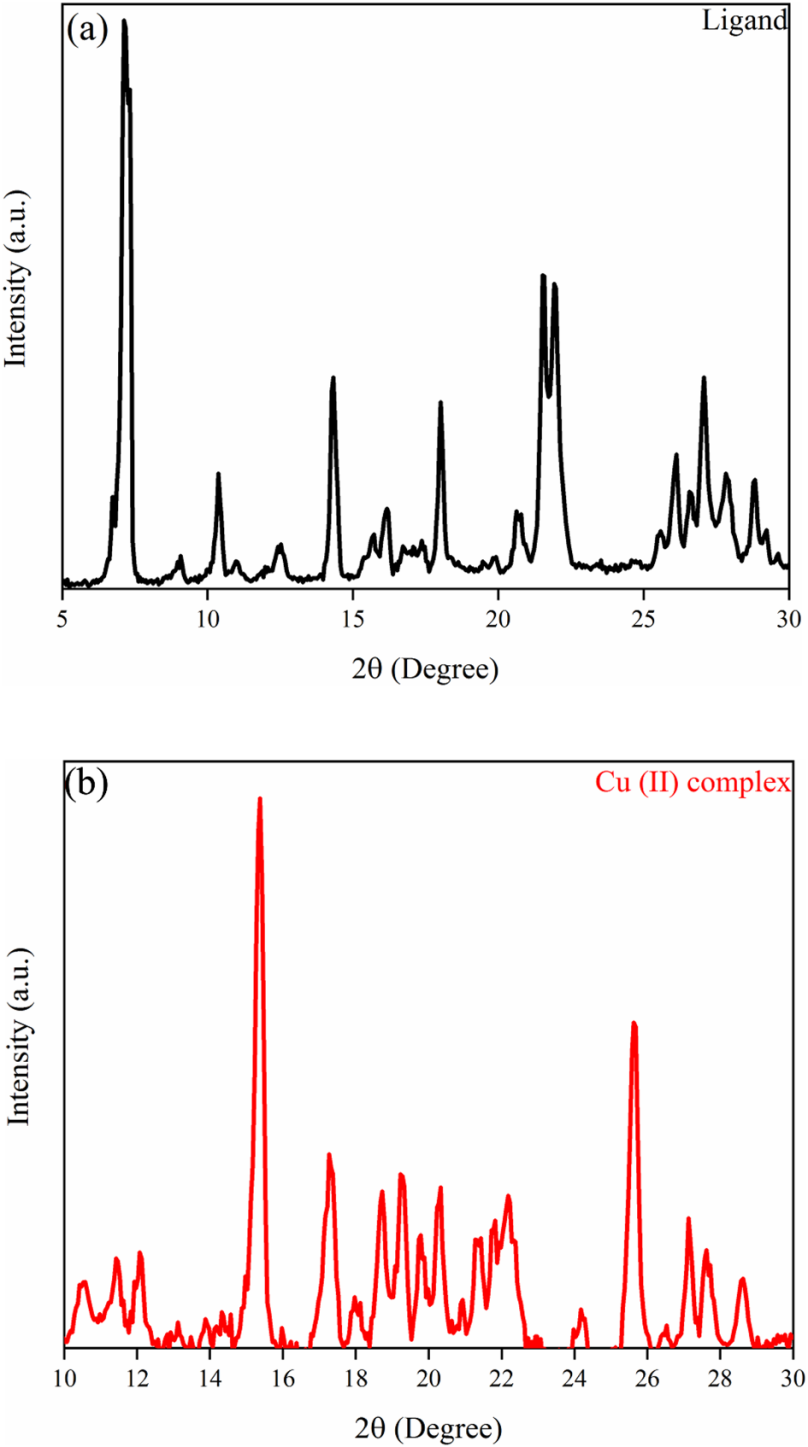
XRD patterns of the (**a**) Ligand, (**b**) Cu (II) Complex.

Figure 2a and b exhibit the diffraction peak patterns of the ligand and its complex with Cu (II) obtained from the X-ray diffraction (XRD) technique. The polycrystalline nature of the synthesized samples is confirmed by the existence of different diffraction peaks in the patterns in Fig. 2. In addition, it can conclude that the synthesized samples have monoclinic structures, which are in good accordance with the reference JCPDS card numbers #96-221-4146 and #96-222-4482 for ligand and its complex with Cu (II), respectively. The three prominent, intense, and characteristic peaks of the synthesized ligand located at 2θ = 7.30°, 14.31°, and 21.52°, which were indexed by their (001), (002), and (003) indices, respectively. Also, for Cu (II) complex, the amounts as mentioned earlier are 2θ = 15.38°, 17.33°, and 25.65°, which were indexed by their (002), (200), and (140) indices, respectively.

By applying Debye–Scherrer relation in XRD data for three prominent peaks, the average crystallite size was calculated^39–41^:

1 $$ {\text{D}} = \frac{{{\text{k}}\uplambda }}{{\upbeta_{{{\text{hkl}}}} {\text{cos}}\uptheta }} $$

Here θ is the angle of the diffraction, λ is the wavelength of the X-ray, β (in radians) is the FWHM and indicates the broadening of the diffraction peaks intensity obtained at half of their maximum, D is the mean crystallite size, and K for Cu-K $\alpha$ equals to 0.9 and is a constant.

Moreover, the dislocation density or the number of defects (δ) is expressed as follows^42^:

2 $$ \delta = \frac{1}{{D^{2} }} $$

The calculated δ values were 8.80 $\times 10$ ^14^ line/m^2^ and 6.25 $\times 10$ ^14^ line/m^2^ for ligand and Cu (II) complex, respectively. Compared to ligand, in the complex_,_ the defects and vacancies have reduced regarding the decrement in δ. On the other hand, the smaller δ values for the Cu (II) complex confirm decrement in structural disorder or crystal imperfections, which leads to increasing crystallite size^43^. The obtained crystallographic data and structural information of Ligand and Cu (II) Complex including reference code, compound name along with peak position, FWHM, and crystallite size are tabulated in Tables 1 and 2, respectively.

**Table 1:** Crystallographic data of Ligand.

System: monoclinic			Reference code: 96-221-4146		
Compound name: 3,5-Dinitro-N-(tri-2-pyridylmethyl)benzamide^44^					
Peak no.	Peak position 2*θ* (°)	FWHM	(hkl)	Crystallite size D (nm)	Avg. D (nm)
1	7.2957	0.3444	001	24.15	33.70
2	14.3093	0.246	002	34.01	
3	21.5234	0.1968	003	42.93

**Table 2:** Crystallographic data of Cu (II) Complex.

System: monoclinic			Reference code: 96-222-4482		
Compound name: Bis(μ-2,2′-biimidazole-κ2 N 3:N 3′) bis­[aqua­copper(I)] sulfate^45^					
Peak no.	Peak position 2*θ* (°)	FWHM	(hkl)	Crystallite size D (nm)	Avg. D (nm)
1	15.3769	0.1968	002	42.56	39.98
2	17.3342	0.2460	200	34.13	
3	25.6503	0.1968	140	43.26	

### Rietveld studies

**Figure 3:**
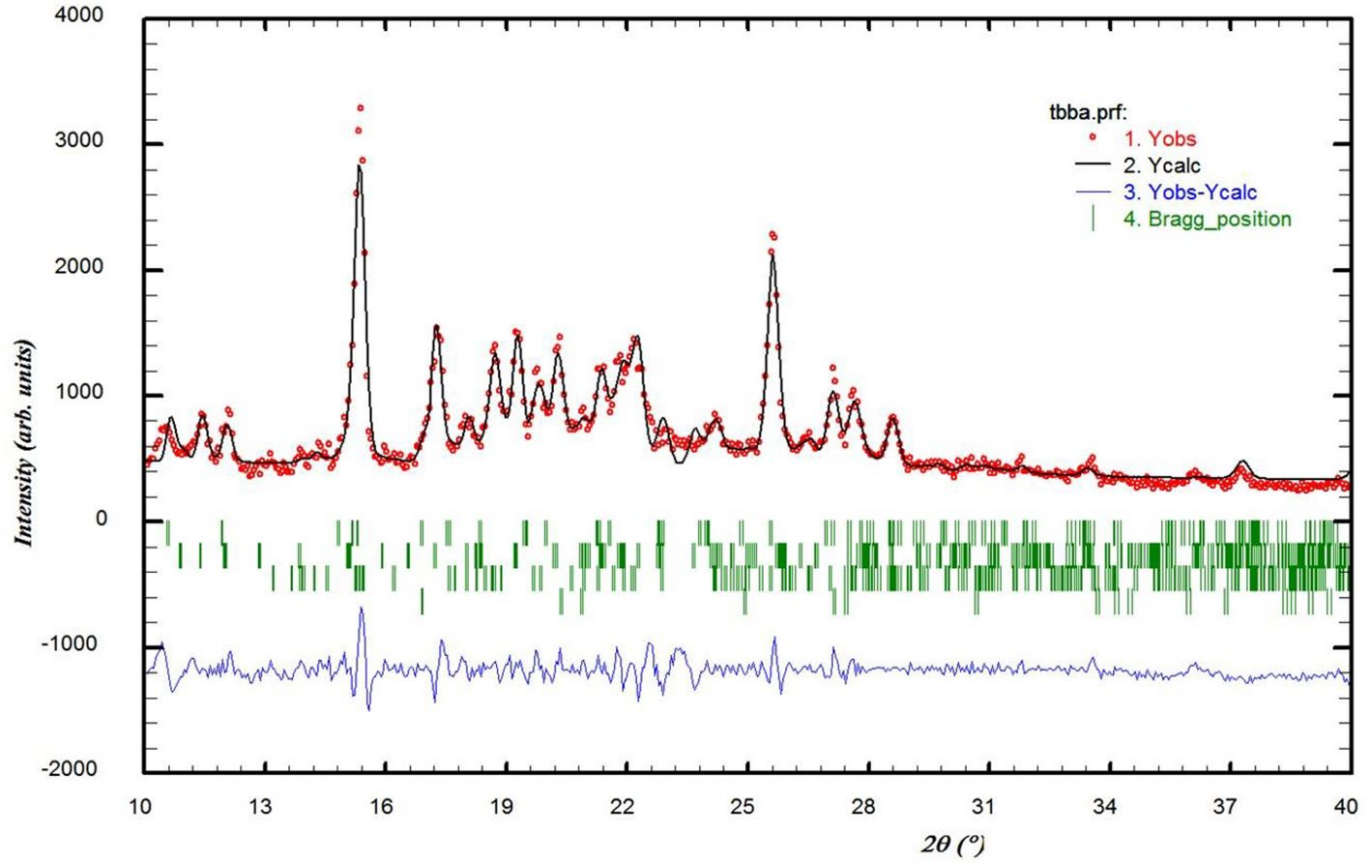
XRD patterns of the Cu (II) Complex. XRPD patterns associated with Rietveld analysis for the produced complex.

The Rietveld technique was used with the *FullProf* software program Version 7.30. Rietveld analysis data confirmed the production of the described complex. The red dots are the observed intensities, while the black lines are the calculated data by obtained Rietveld analysis. The Bragg reflection positions are indicated by green bars, which correspond to the crystal phases. The number of bars rows shows the number of the crystal phases. The blue line under the green bars is the difference between the observed and calculated data. Figure 3 shows that the product is a mixture of 4 crystal phases (green bars). The bars from top to down correspond to the synthesized complex, ligand (S_1_), ligand (S_2_), and CuSO_4_, respectively. By comparing the peak intensities of the phases in the product mixture, it shows that the main phase is obtained for the complex (Reference code: 96-222-4482). However, according to the explanations included in the “Supplementary file”, the raw materials (ligand and CuSO_4_) exist in the product mixture and so a composite product has been obtained.

### FESEM Analysis

**Figure 4:**
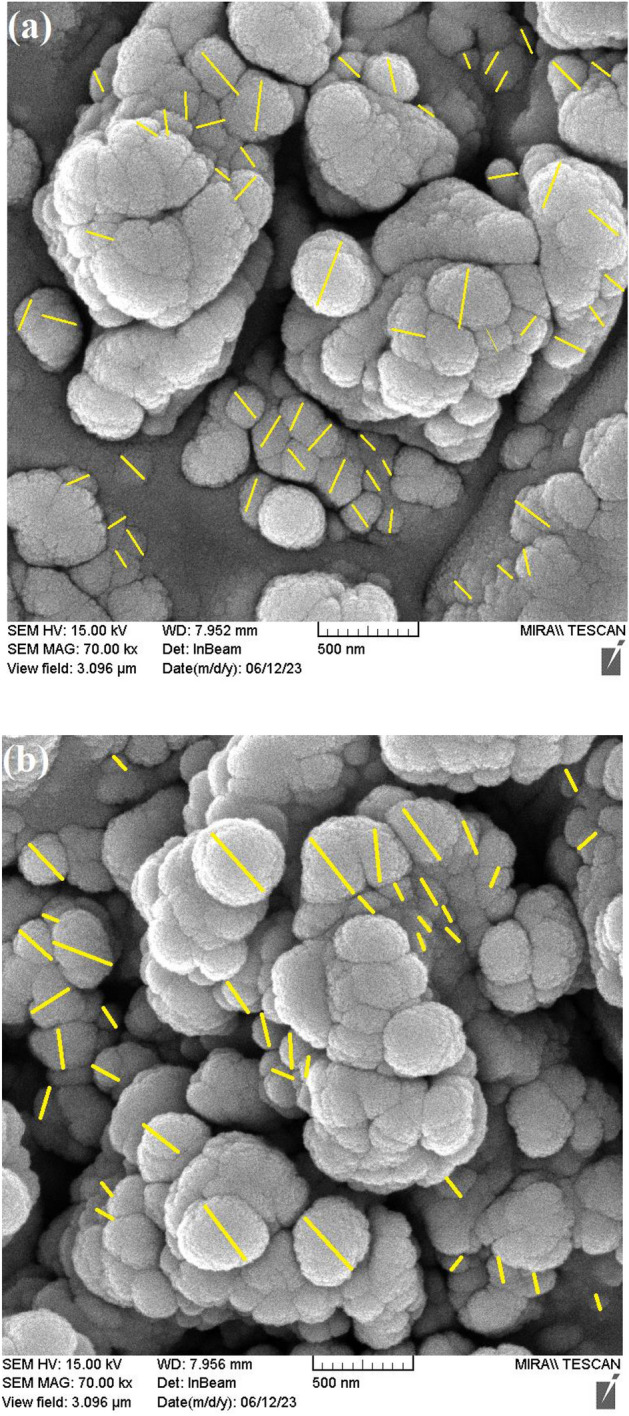
FESEM image of (**a**) Ligand and (**b**) Cu(II) Complex.

**Figure 5:**
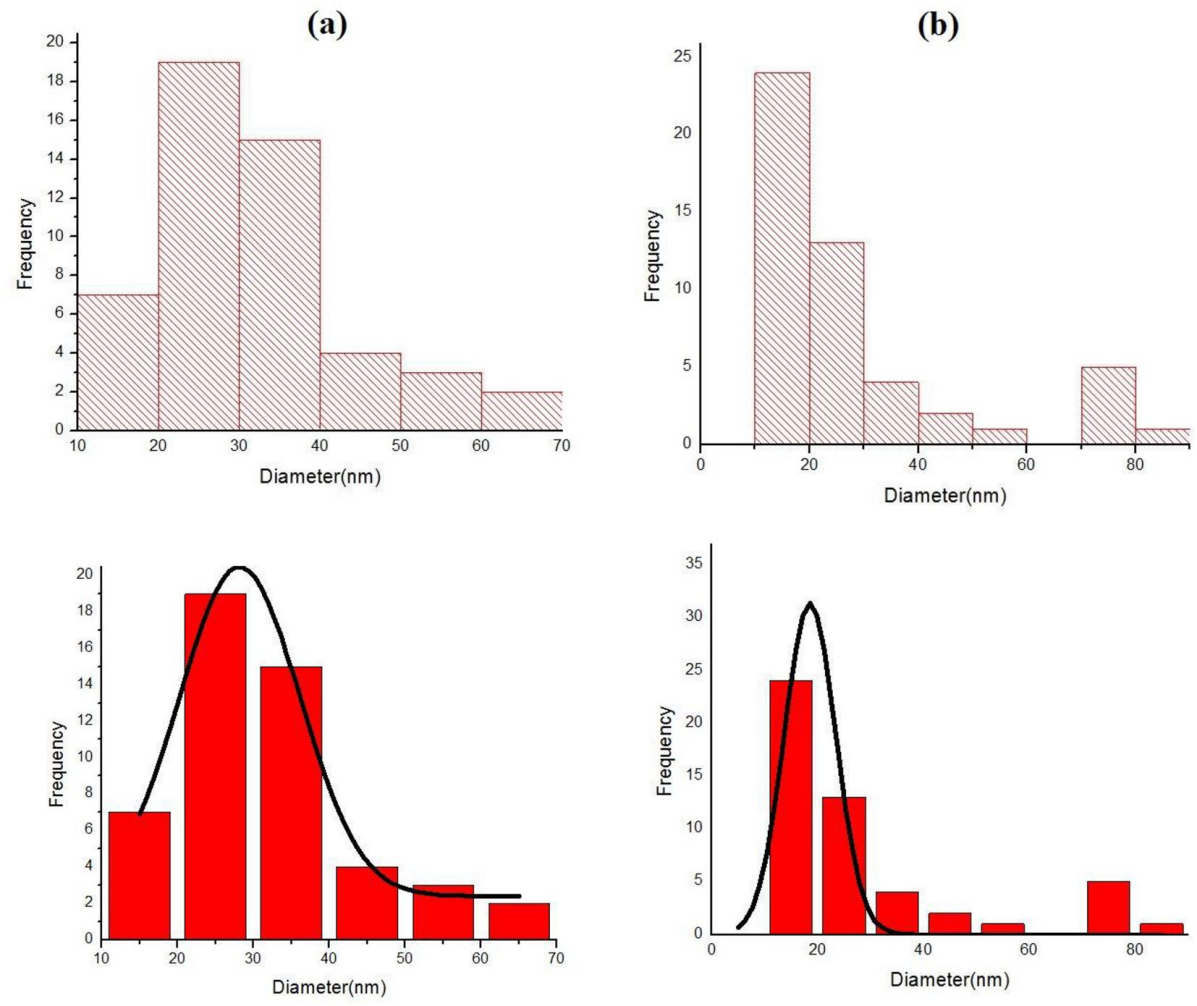
Particle size distribution histogram of (**a**) Ligand and (**b**) Cu(II)Complex.

To investigate the surface morphology ligand and Cu (II) complex, FESEM analysis was used and the representative images of various samples are depicted in Fig. 4a,b. Particle size and the morphology of FESEM micrographs indicated that in good agreement with the calculated result from the Debye–Scherrer equation. Furthermore, the particle size distribution histogram of the synthesized samples is depicted in Fig. 5a,b.

### EDX analysis

**Figure 6:**
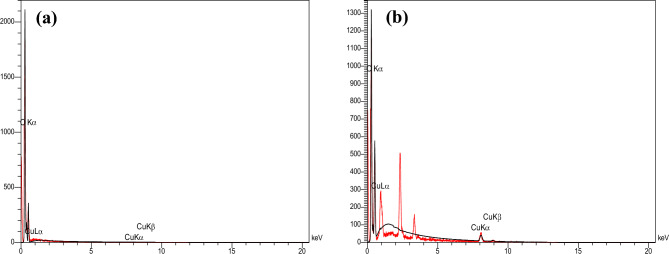
EDX analysis of (**a**) Ligand and (**b**) Cu(II)-Complex.

**Figure 7:**
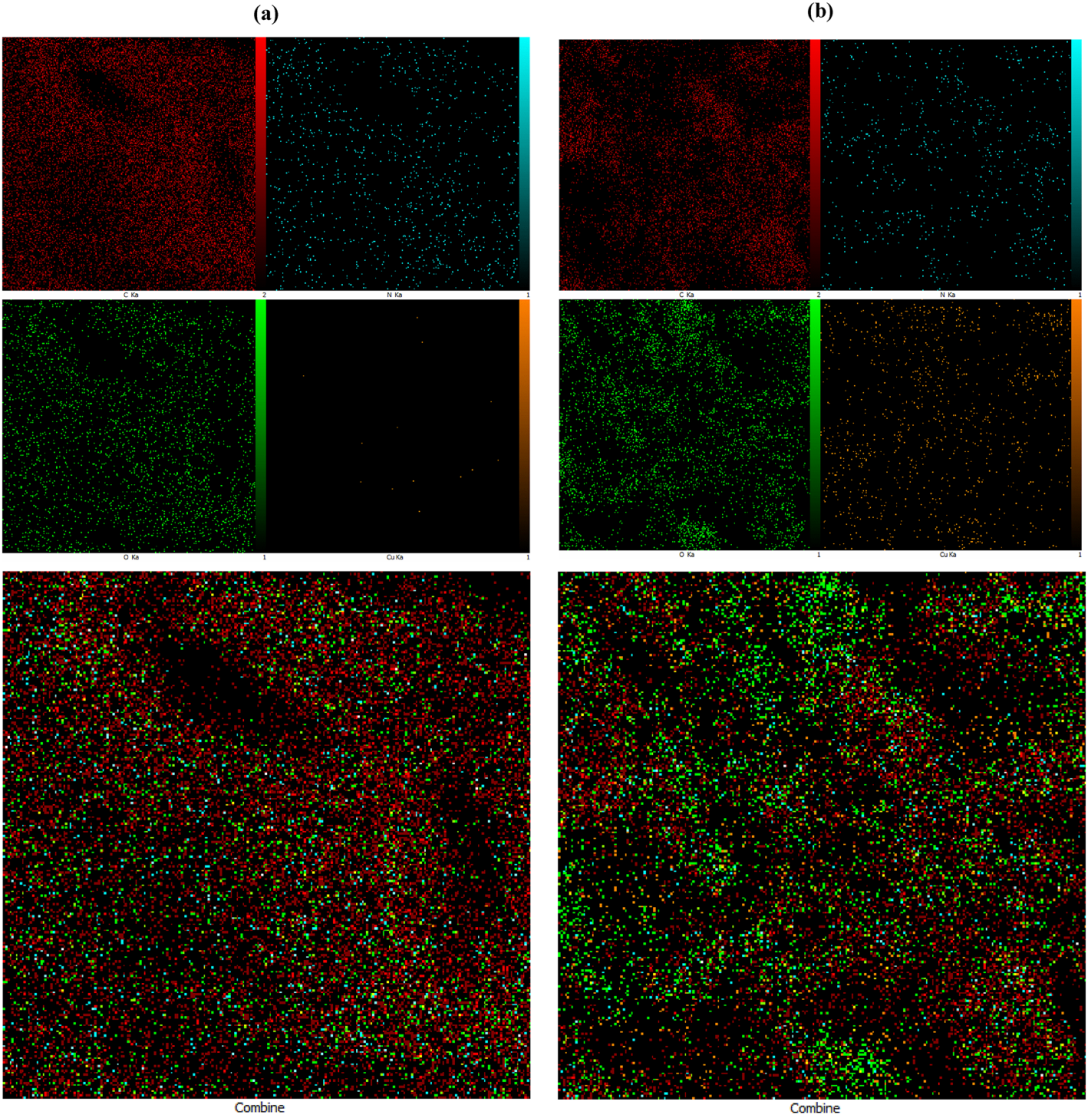
Elemental mapping of (**a**) Ligand and (**b**) Cu (II)-Complex.

To verify the existence of applied components as well as the distribution of Cu (II) in the Complex structure, the EDX dot-mapping analysis was carried out. The EDX dot-mapping micrographs of the Ligand and Cu(II)-Complex have been depicted in Fig. 6a,b and 7a,b.

### ^1^***H*** and ^13^***C*** NMR spectra

**Figure 8:**
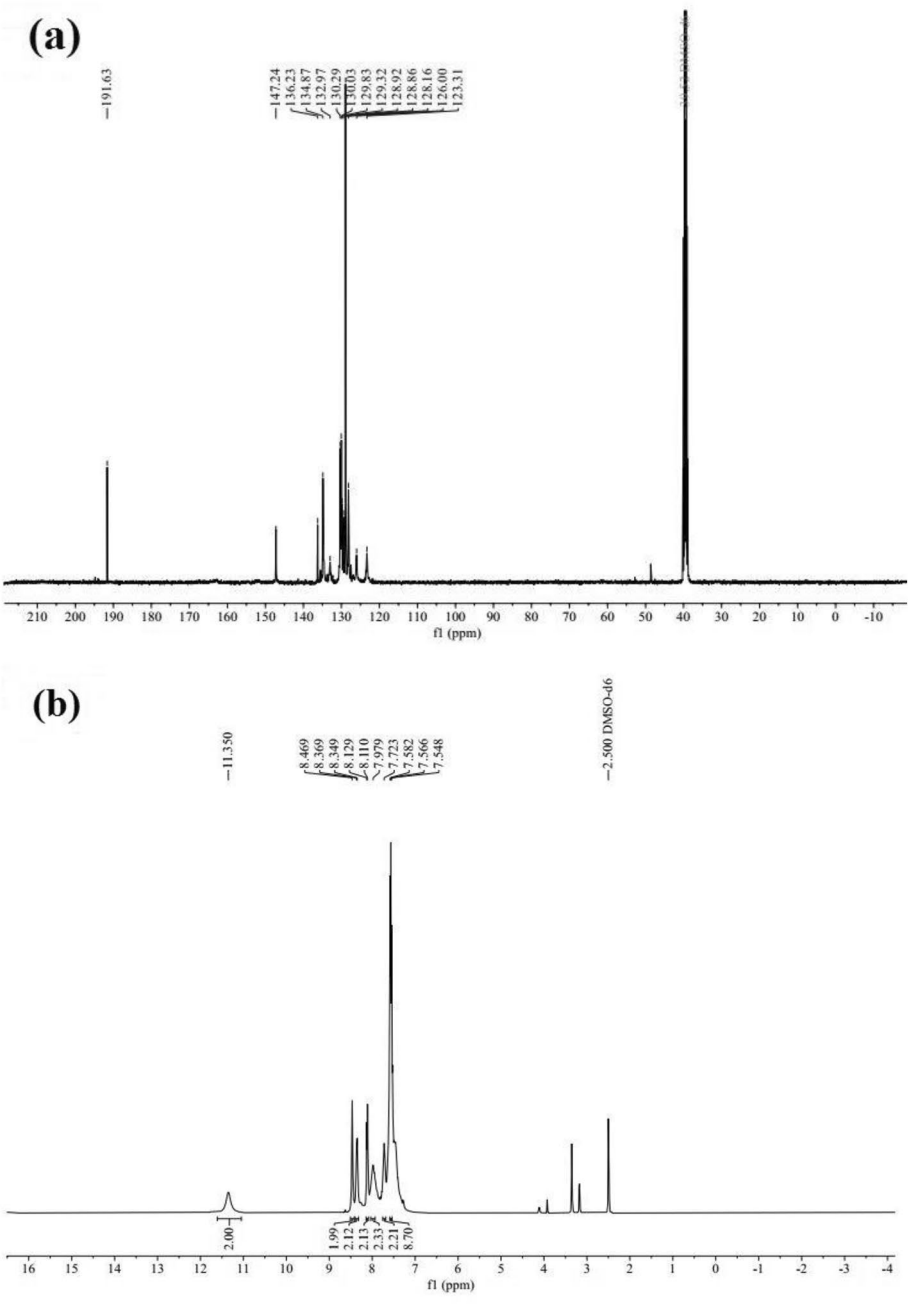
(**a**) ${}^{13}C$ NMR and (**b**) ${}^{1}H$ NMR spectrum of the ligand.

Figure 8 demonstrates the ${}^{1}H$ NMR and ${}^{13}C$ NMR spectra of the synthesized ligand.

**Figure 9:**
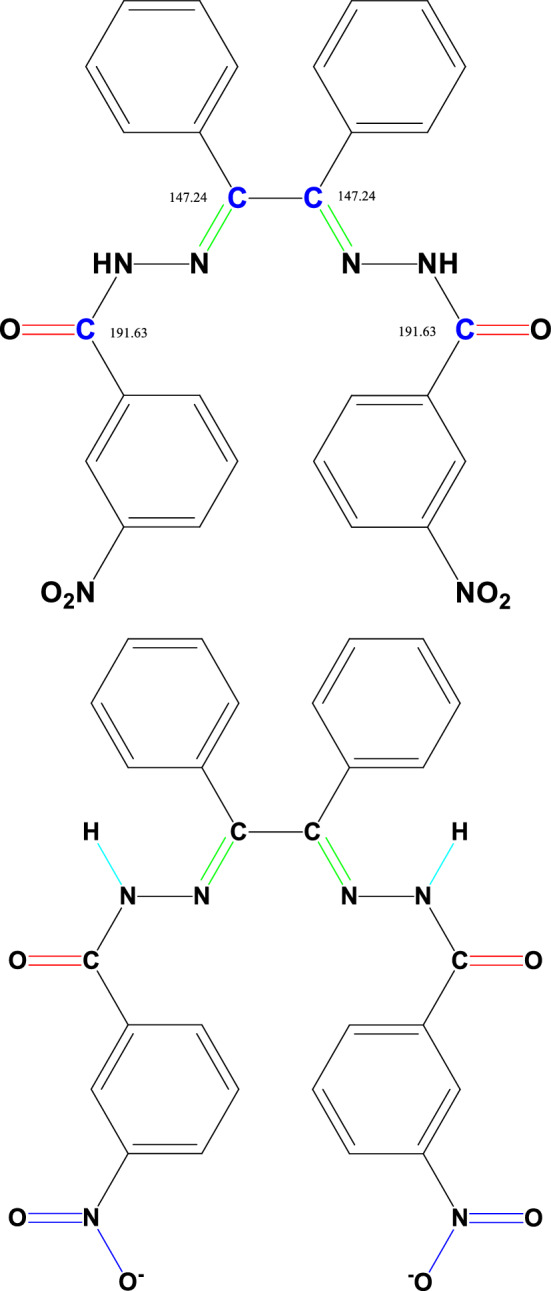
Structures of the Carbonyl and Imine groups.

In Fig. 8a, which demonstrates the ${}^{13}C$ NMR spectra of the ligand, the observed peaks in a chemical shift at 191.63 ppm and 147.24 ppm are associated with the carbon of the carbonyl and imine groups (Fig. 9).

**Figure 10:**
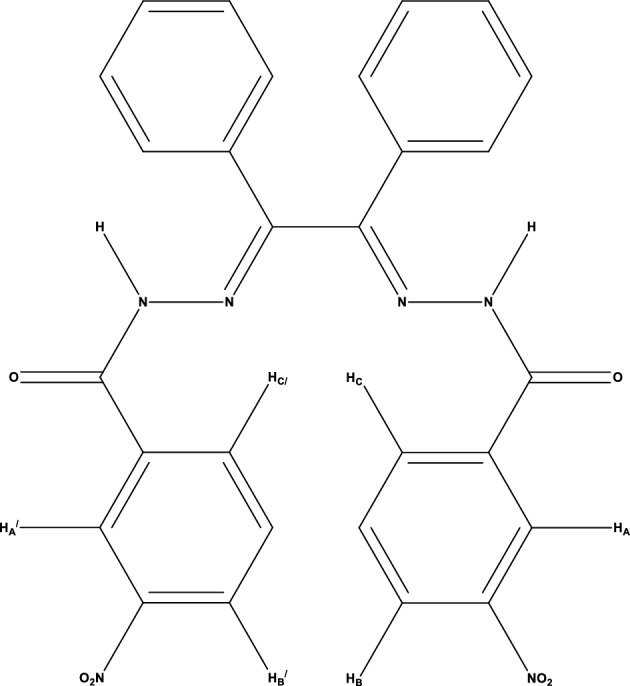
Structure of the 3-nitrophenyl rings.

On the other hand, in the ${}^{1}H$ NMR spectrum shown in Fig. 8b, the observed broad singlet (bs) peak in the 11.35 ppm area could be associated with NH. In addition, the observed sharp singlet (s) peak in the $\delta$ = 8.47 ppm area may be related to $H_{A}$, $H_{A^{\prime}}$ hydrogens in the 3-nitrophenyl ring. The doublet (d) peak at $\delta$ = 8.36 ppm (2H, d, *J* = 8 Hz, ArH) belongs to the $H_{B}$, $H_{B^{\prime}}$ protons in the 3-nitrophenyl ring. Also, another doublet peak at $\delta$ = 8.12 ppm (2H, d, *J* = 7.6 Hz, ArH) was attributed to the $H_{C}$, $H_{C^{\prime}}$ hydrogens in the 3-nitrophenyl ring (Fig. 10).

According to Fig. 8, the full ${}^{13}C$ NMR and ${}^{1}H$ NMR assignments of the synthesized ligand are listed in Table 3.

**Table 3:** ${}^{13}C$ NMR and ${}^{1}H$ NMR spectral data of the ligand.

^13^C NMR Data (*δ*, ppm): (100 MHz, DMSO-*d*_*6*_)	123.31, 126.00, 128.16, 128.86, 128.92, 129.32, 129.83, 130.03, 130.29, 132.97, 134.87, 136.23, 147.24, 191.63
^1^H NMR Data (*δ*, ppm): (400 MHz, DMSO-*d*_*6*_)	7.54–7.58 (8H, m, ArH), 7.72 (2H, s, ArH), 7.79 (2H, s, ArH), 8.12 (2H, d, *J* = 7.6 Hz, ArH), 8.36 (2H, d, *J* = 8 Hz, ArH), 8.47 (2H, s, ArH), 11.35 (2H, bs, NH);

m = multiplet, d = doublet, s = singlet, bs = broad singlet.

### Functional studies (Fourier transform infrared spectroscopy (FT-IR))

**Figure 11:**
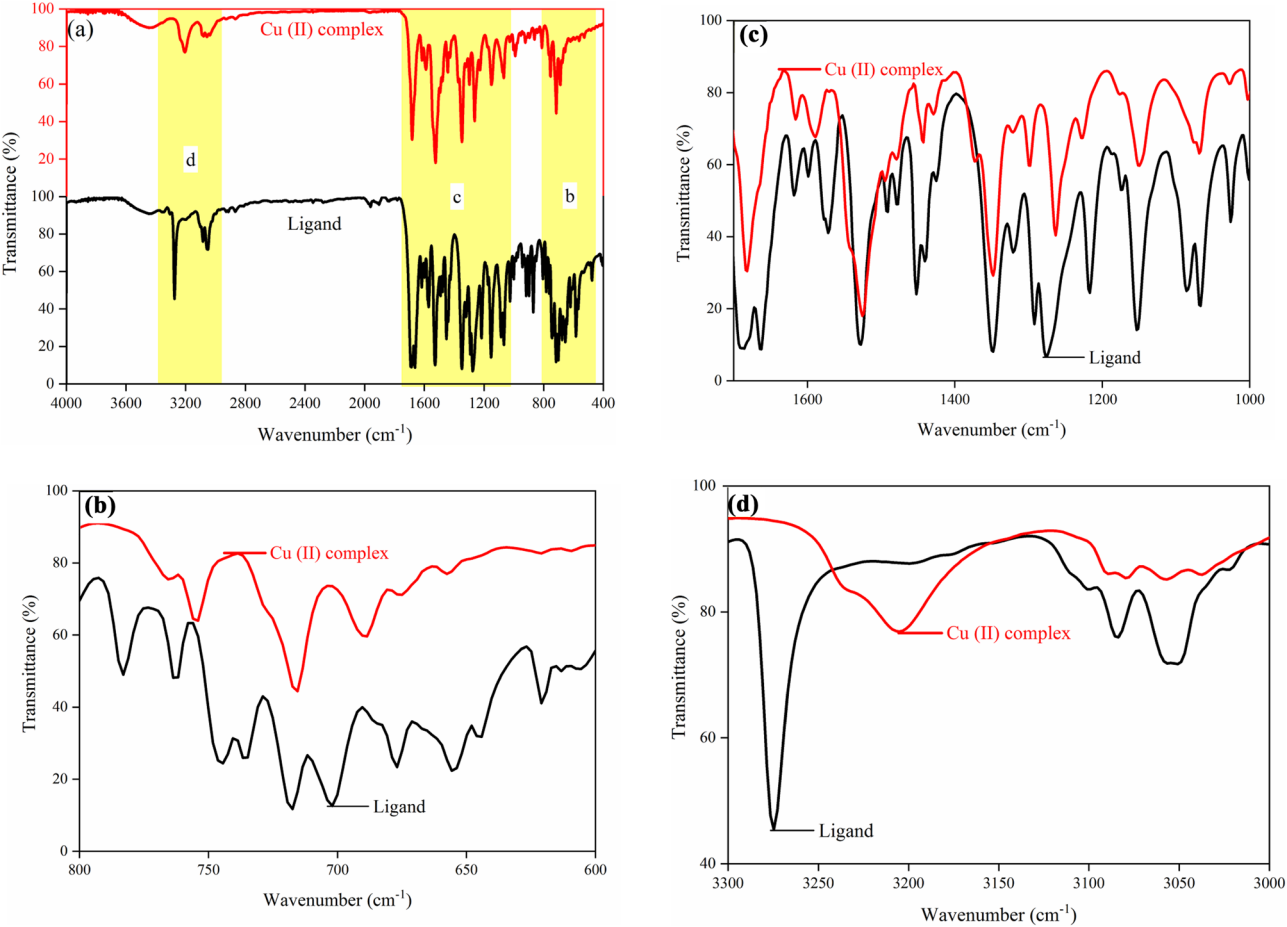
FT-IR spectra of the Ligand and Cu (II) complex. (**a**) in the range of 400–4000 cm^−1^, (**b**) in the range of 600–800 cm^−1^, (**c**) in the range of 1000–1700 cm^−1^, and (**d**) in the range of 3000–3300 cm^−1^.

**Figure 12:**
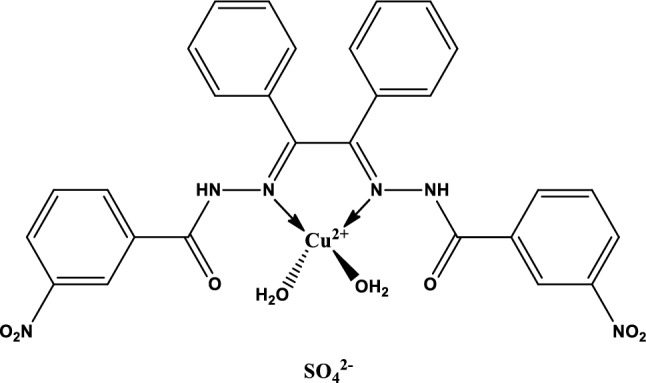
Structure of the nitro groups (N–H stretching vibrations).

To evaluate the Sb ligand bonding mode to Cu ion, we studied the ligand and its complex FT-IR spectrum in the range of 400–4000 cm^−1^. The obtained FT-IR spectra for ligand and Cu (II) Complex are exhibited in Fig. 11a–d in various wavenumber ranges. Peaks at 3274 cm^−1^ and 3205 cm^−1^ are assigned to the N–H stretching vibrations. These stretching vibrations at vibrational peaks in the synthesized samples determine the hydrogen bond^46^. The absorption band of the amide carbonyl moves toward the lower frequencies due to conjugation with an aromatic ring and appears at 1682 cm^−1^ and 1686 cm^−1^ for Cu (II) Complex and ligand, respectively. The absorption band of the imine group is observed at 1663 cm^−1^. Peaks at 1528 cm^−1^, 1525 cm^−1^, and 1348 cm^−1^ could be linked to symmetry and asymmetry stretching absorption of the nitro groups (Fig. 12). The C–H stretching vibrations are ascertained in the 3150–2900 cm^−1^ region. On the other hand, in the synthesized Cu (II) Complex sample, the broad and small peak around 3450 cm^−1^ expressed coordinated and crystalline water^47^. The Cu–O stretching vibration mode was observed in the 590–520 cm^−1^ region^48^. Table 4, is represented the fundamental FT-IR spectral bands for Cu (II) Complex and ligand.

**Table 4:** FT-IR spectral bands (cm^−1^).

Wavenumber (cm^−1^)		Assignments
Ligand	Cu (II) complex	
3274 (s)-3053 (b)	3205 (b)-3058 (w)	N–H stretching vibrations^49^
3084 (m)	3079 (sh)	C–H stretching vibrations^50^
2861 (w)	2867 (w)	C–H stretching^23^
1686 (sh)	1682 (s)	C=O^51^
1619 (w)-1598 (w)	1616 (w)-1589 (w)	C=N stretching^52,53^
1528 (s)	1525 (s)	N–H bending^53^
1452 (sh)	1442 (sh)	C=C stretching^54^
1216 (m)	1226 (w)	C–N stretching^52^
1066 (sh)	1067 (b)	C–N and N–N stretching^23,51^
1024 (s)	1027 (w)	Oxygenated functional epoxy groups^55^
762 (w)	754 (w)	C–H^56^

*b* broad, *sh* shoulder, *w* weak, *m* medium, *s* strong.

### Magnetic properties

**Figure 13:**
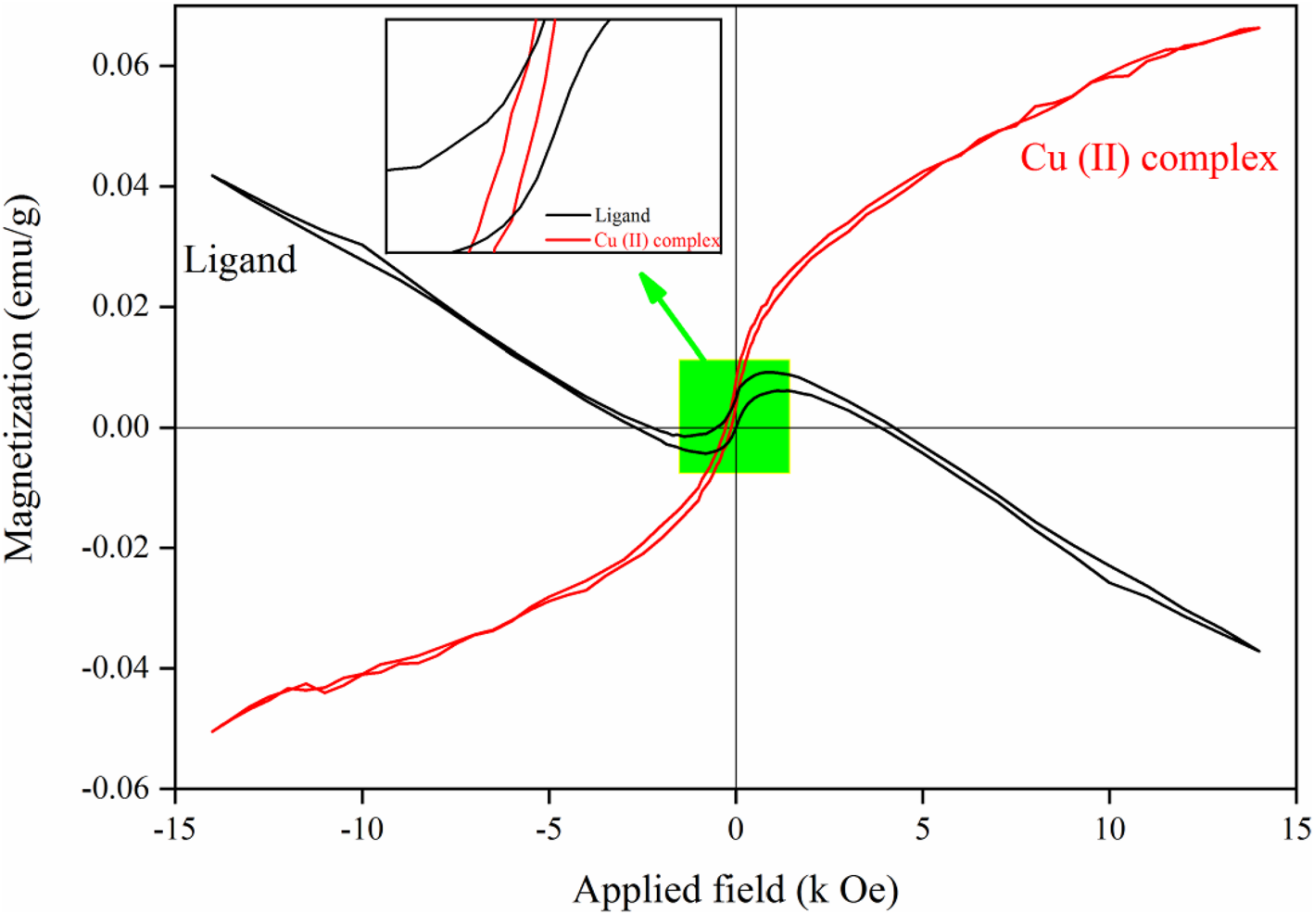
Magnetic curves of the Cu (II) Complex and ligand.

Recently, investigating the magnetic properties of transition metal polynuclear complexes with Schiff base ligands has attracted enormous interest. These compounds have properties of single-molecule magnetism and single-chain magnetism and are used as a precursor for molecular magnetic materials^57^. In the past decades, Cu (II) complexes achieve great interest because of their important role in the field of molecular magnetic^58–62^. The selection of metal ions with suitable Schiff base ligands for bridging between metal ions in multinuclearity complexes is an important factor in causing magnetic behavior in a complex^63^. A vibrating sample magnetometer (VSM) is utilized to evaluate the synthesized samples magnetically. Figure 13 demonstrates the recorded hysteresis loops (M-H curves) of ligand and Cu (II) Complex under a range of $\pm$ 15 kOe magnetic field at room temperature. As can be observed, the synthesized Cu (II) Complex sample indicates a dominant paramagnetic phase that is not magnetically saturated even at 14 kOe applied field, which could be attributed to the paramagnetic nature of the Cu(II)^64^. Also, it has a weak ferromagnetism phase, and its extracted data were summarized in Table 5. On the other hand, for the ligand, we can observe a diamagnetic phase. The saturation magnetization $\left( {M_{s} } \right)$of the Cu (II) Complex and ligand were 0.066 and 0.042 emu/g, respectively. The $M_{s}$ value of the Cu (II) Complex was higher in comparison to the ligand. Compared to ligand, the remanence magnetization $\left( {M_{r} } \right)$and coercive field $\left( {H_{c} } \right)$values for the Cu (II) Complex demonstrated an increment. As a result, higher magnetization in the Cu (II) Complex could originate from an increment in the crystallite size, which leads to enhancement in magnetic ordering^65^. These results represent a good accordance between structural and magnetic properties. Moreover, the appearance of the Cu phase in the synthesized Cu (II) Complex sample (as observed in the XRD patterns) affected the magnetic characteristics and changed them. As mentioned above, variation in crystallite size is a critical point that leads to $M_{s}$ variation^66^. To investigate the magnetic hardness and domain nature of the samples, a critical property is introduced named squareness ratio $\left( {K_{P} } \right)$expressed as $M_{r} /M_{s}$ ratio. If the $K_{P}$ is higher than 0.5, it expresses a material with a single domain, is highly anisotropic, and is magnetically hard. Visa versa, if the K_p_ is lower than 0.5, the material is randomly oriented and multi-domain^67^. In addition, $H_{c}$ is characterized as a magnetic hardness. Materials falling within the range of $10^{3} {\text{Am}}^{ - 1} \left\langle {H_{c} } \right.\left\langle {10^{4} } \right.{\text{Am}}^{ - 1}$ are classified as soft materials^68^. According to our calculations on Cu (II) complex, the value of $H_{c}$ is approximately equal to 9 × $9 \times 10^{3} {\text{Am}}^{{{ - }{1}}}$, which implies that this sample is a soft room temperature ferromagnetism (RTFM) material. Additionally, the low $K_{P}$ and $H_{c}$ values provide further evidence of the soft ferromagnetic nature of the Cu (II) complex. The magnetic information of the Cu (II) Complex and Ligand samples were summarized in Table 5.

**Table 5:** The magnetic parameters of the Cu (II) Complex and ligand.

Sample	$M_{r}$(emu/g)	$M_{s}$(emu/g)	$H_{c}$(Oe)	Loop Area (Oe $\times$ emu/g)	$K_{p}$
Ligand	0.005	0.042	2.85	41.221	0.11
Cu (II) Complex	0.008	0.066	125	14.217	0.12

### DFT study

**Figure 14:**
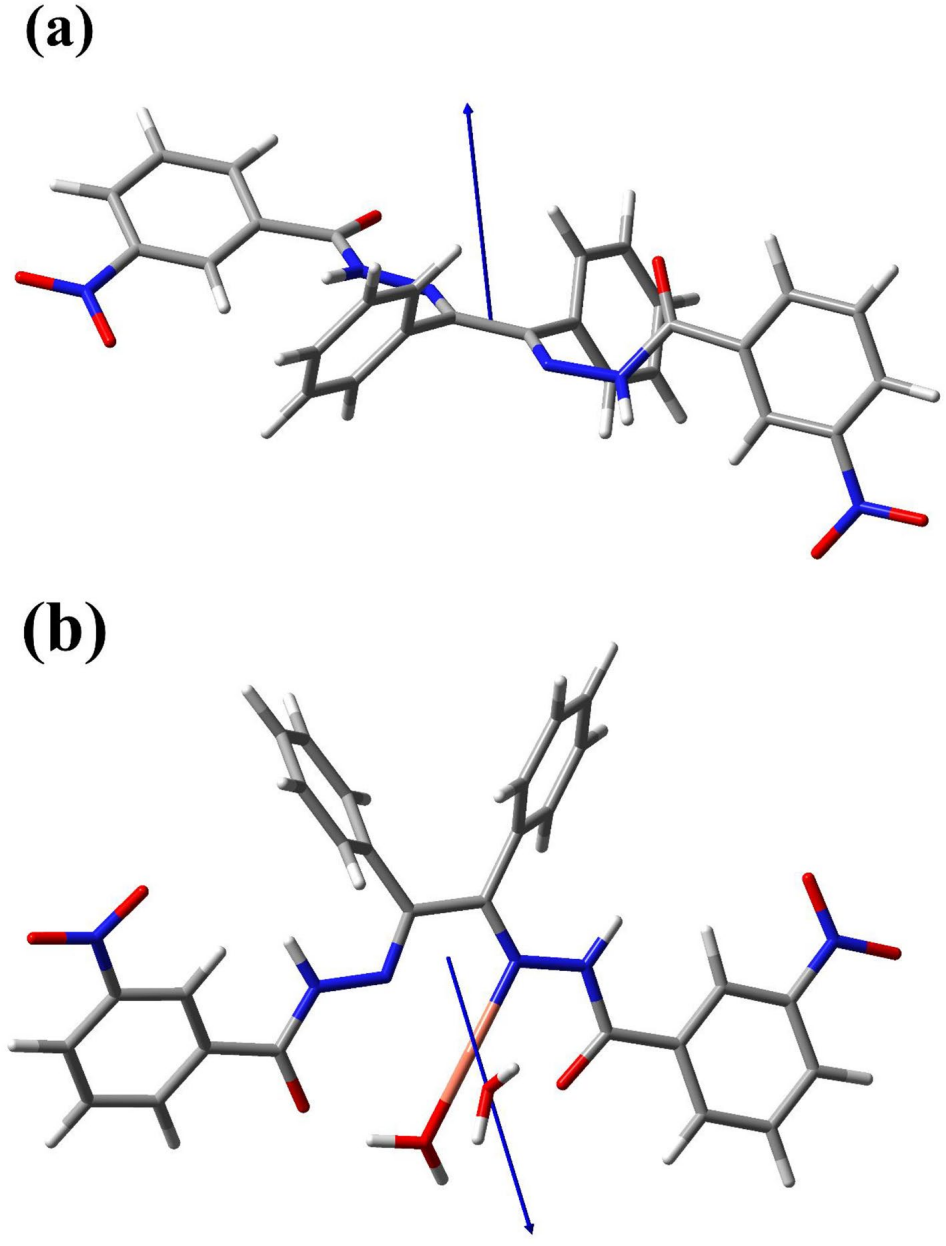
The optimized structure of the (**a**) Ligand; (**b**) Cu (II) Complex.

In both synthesized samples all obtained vibrational frequencies are positive which confirms that the samples are stable energetically and the optimized structures are correct. Then the dipole moment of each molecule was calculated. The structure of the molecules and diple moment vector of the Ligand and Cu (II) Complex is illustrated in Fig. 14. The dipole moment of the samples was found to be 4.473 Debye and 5.345 Debye for the Ligand and Cu (II) Complex, respectively, which revealed that the Cu (II) Complex is more polarized than Ligand. In other words, the dipole moment deduced from this study reveals the nature of the intramolecular charge transfer due to the electron-accepting nitro groups. Comparing the dipole moments of ligand and complex, we can conclude that the introduction of the nitro group in the meta position favors intramolecular charge transfer in the direction pointed by the dipole vector. The observed dipole moments suggest that twisting of either the donor (D) concerning the acceptor (A) or vice versa can have a large impact on the resultant charge distribution. Ligand and complex have twisted structures. Also, Ligand and complex show extensive charge transfer. This is partly due to the strong electron-withdrawing power of the $- NO_{2}$ group. The low polarity of ligand results mainly from the electronic competition of the two electron-accepting groups. These dipolar fragments are oriented in opposite directions, as expected.

### Linear and nonlinear optical studies

#### Linear optical properties

**Figure 15:**
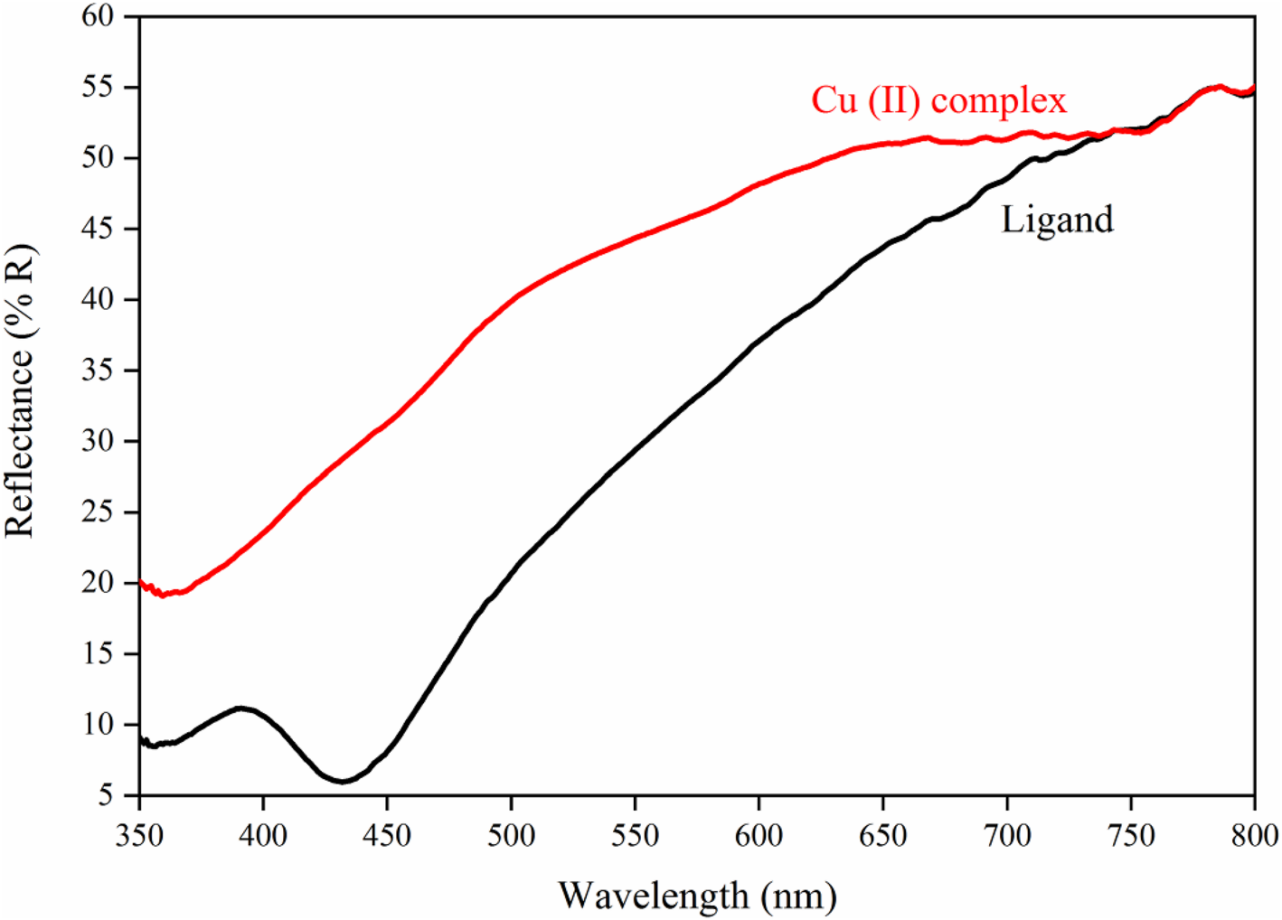
Reflectance spectra of the Cu (II) Complex and Ligand.

**Figure 16:**
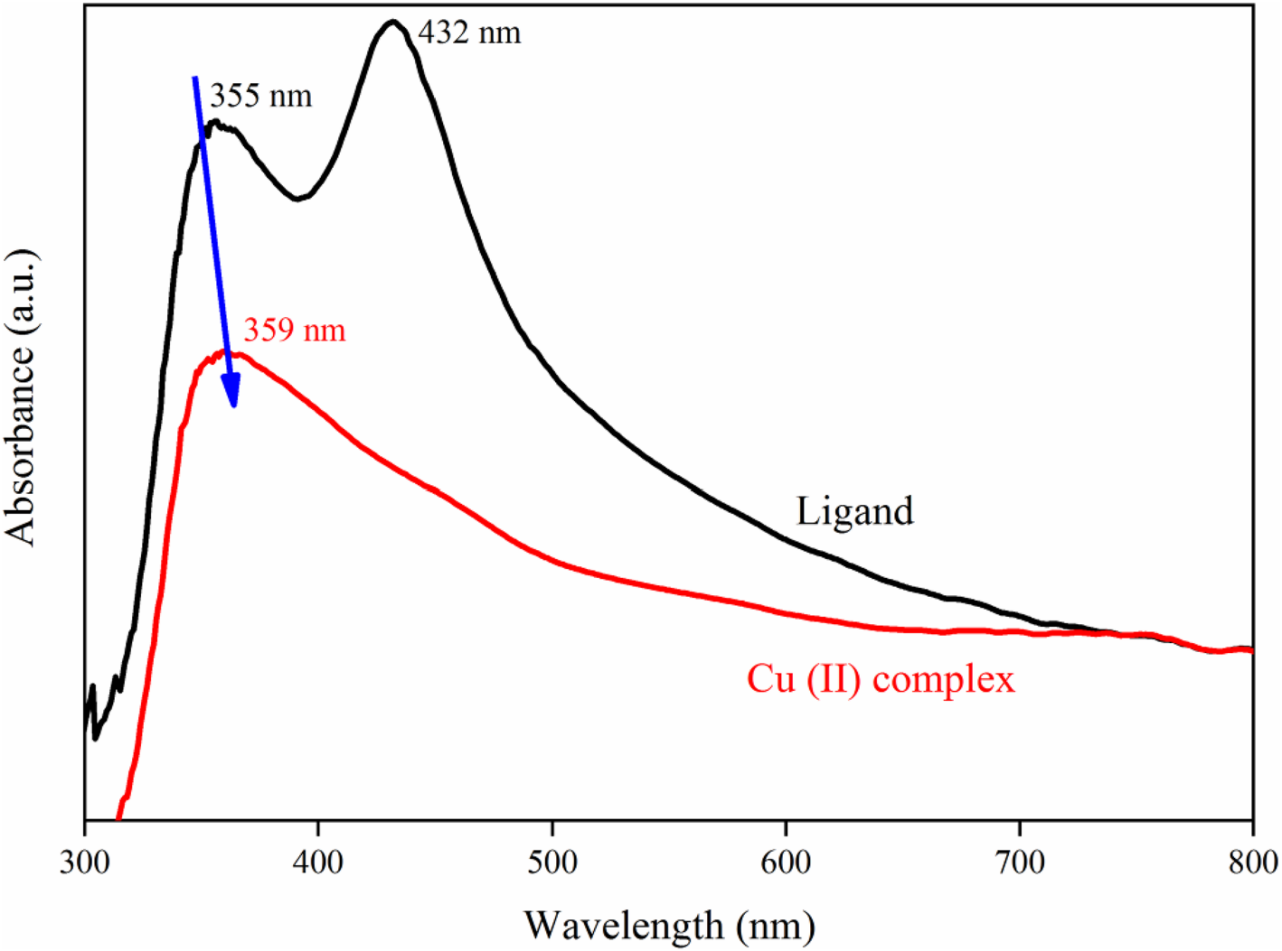
Absorbance spectra of the Cu (II) Complex and ligand.

The optical absorption and reflectance and spectra of the Cu (II) Complex and Ligand obtained from Diffuse Reflectance Spectroscopy (DRS) recorded in the wavelength range of approximately 300–800 nm are plotted in Figs. 15 and 16. As observed in Fig. 15, Cu (II) Complex has higher reflectance values compared to the ligand. Moreover, some significant peaks appeared around 350–450 nm because of the transition between conduction and valence bands. The absorption decrement in the UV–visible area could originate from transitions consisting of extrinsic states like existing impurities, defect states, or surface traps^69^. As can be observed in Fig. 16, two electronic transitions are displayed at 355 nm and 432 nm for the ligand, which could be related to the n → π^∗^ or π → π^∗^ transitions that determine charge transfers at intra-ligand (IL). Besides, the existence of the latter and additional transition at 432 nm represents another order of π-electronic conjugation (π-electrons). On the other hand, these IL transitions in Cu (II) complex can observe at 359 nm, which, in comparison to the ligand, it expressed lower intensities and a redshift^70^. The monoclinic phases and highly crystalline nature of the synthesized samples are verified by the first sharp rise, in the absorbance spectra below 360 nm^71^.

Equation (3) initially introduces the Kubelka–Munk theory of reflectance spectroscopy and in the next step, expresses the Tauc relation to obtaining the band gap energies of the synthesized samples as follows^72,73^:

3 $$ F(R) \propto \frac{{\left( {1 - R} \right)^{2} }}{2R} = \frac{K}{S} \propto \alpha \propto \frac{{\left( {h\upsilon - E_{g} } \right)}}{h\upsilon }^{1/n} $$

Here K and S are the absorptions and scattering coefficients, respectively, h is Planck’s constant, n is equal to 1/2 for directly allowed transitions, and υ is the photon frequency.

**Figure 17:**
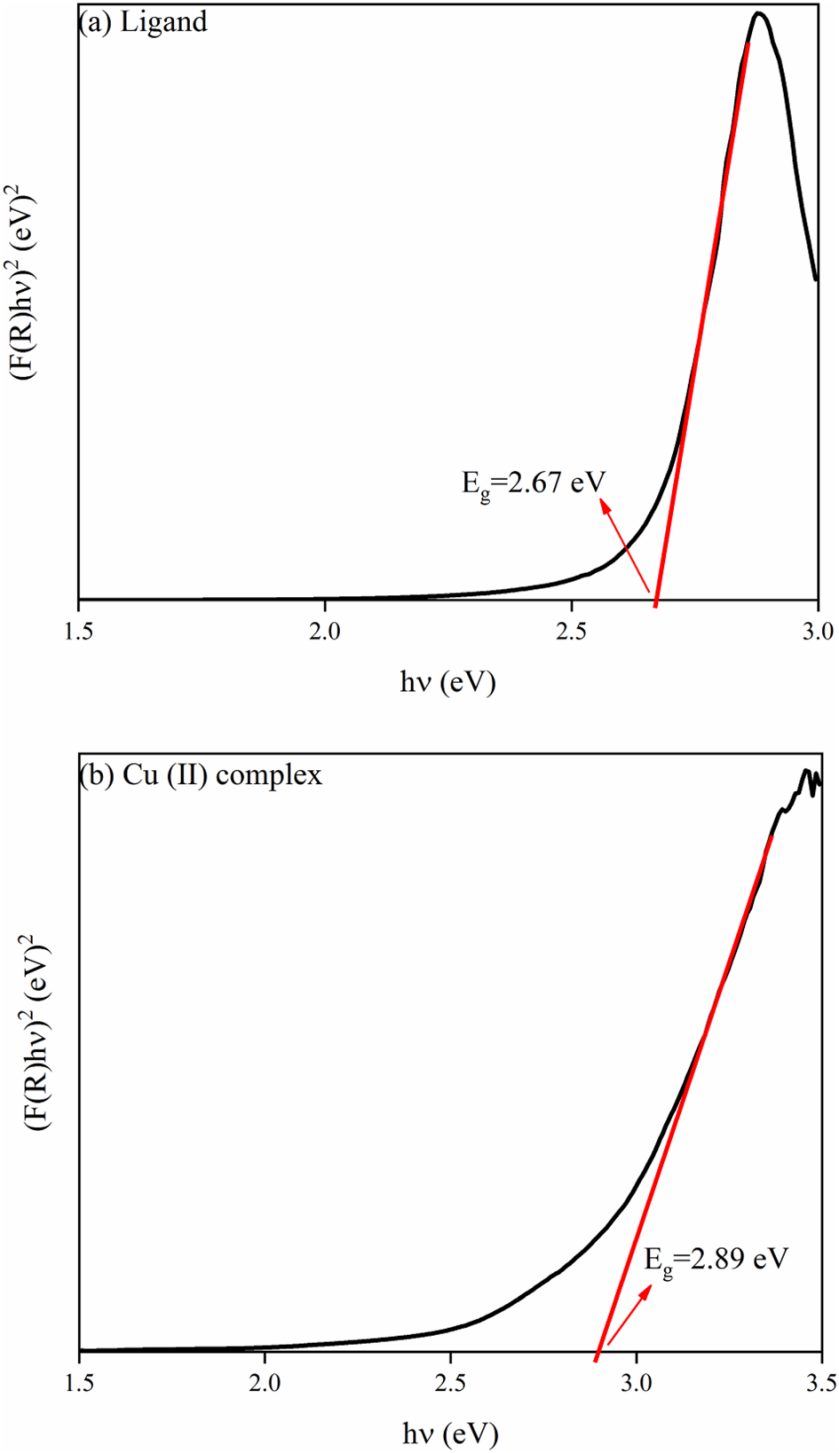
Band gap determination plots for (**a**) Ligand; (**b**) Cu (II) Complex.

Through DRS data and the relation mentioned above, we could estimate the band gap energies of the synthesized samples by plotting the $(F(R)h\upsilon )^{2}$ verses $h\upsilon$ as indicated in Fig. 17a,b extrapolating each plot to zero value $(F(R)h\upsilon )^{2}$. The band gap energies were calculated as 2.67, and 2.89 for ligand and Cu (II) complex, respectively, which point out inter-band transitions originated from permitted direct transitions. The band gap energies chiefly depend on the degree of structural disorder and structural defects of the materials^69^.

Furthermore, due to the low values of the band gap energies, it seems that the synthesized samples to be exhibited optical conductivity properties. Therefore, for conductivity measurements, the Ligand and Cu (II) Complex samples with a concentration of 19 mM alongside a Dimethylformamide (DMF) solvent were measured by a conductometer (Model: 86550, Brand: AZ(EC/TDS)—Taiwan)^74^. The obtained results were obtained to be 1.5 µSm^−1^, 13.7 µSm^−1^, and 102 µSm^−1^ for DMF, Ligand, and Cu (II) Complex, respectively.

#### Kramers–Kronig studies

The determination of the optical parameters, namely the refractive index (n) and extinction coefficient (k), is a crucial task in the realm of filters, optical devices, and optoelectronic switches^75^. Among the various methods employed for the calculation of optical constants, the Kramer-Kronig (K-K) method, with the aid of MATLAB programming, has proven to be useful for this purpose. The complex refractive index ${\text{n}}^{ * } (\omega )$ is of paramount importance in the analysis of optical properties and may be expressed in the following relation^76^:

4 $$ {\text{n}}^{ * } (\omega ) = {\text{n}}(\omega ) + {\text{ik}}(\omega ) $$

By utilizing the K-K relation on reflectance data, the values of k and n can be determined through Eqs. (5) and (6), respectively.

5 $$ {\text{n(}}\omega {)} = \left( {\frac{{{1} - {\text{R(}}\omega {)}}}{{{1} + {\text{R(}}\omega {)} - {2}\sqrt {{\text{R(}}\omega {)}} {\text{cos}}\varphi {(}\omega )}}} \right) $$

6 $$ {\text{k(}}\omega {)} = \left( {\frac{{{2}\sqrt {{\text{R(}}\omega {)}} {\text{sin}}\varphi {(}\omega {)}}}{{{1} + {\text{R(}}\omega {)} - {2}\sqrt {{\text{R(}}\omega {)}} {\text{cos}}\varphi (\omega )}}} \right) $$

Using K–K dispersion, we can calculate the φ(ω) (phase angle) as follows:

7 $$  \varphi (\omega ) = \left( {\frac{\omega }{\uppi }} \right)\int\limits_{0}^{\infty } {\frac{{{\text{ln R(}}\omega ^{\prime } ) - {\text{ln R(}}\omega )}}{{\omega ^{{\prime 2}}  - \omega ^{2} }}} {\text{d}}\omega ^{\prime }  $$

**Figure 18:**
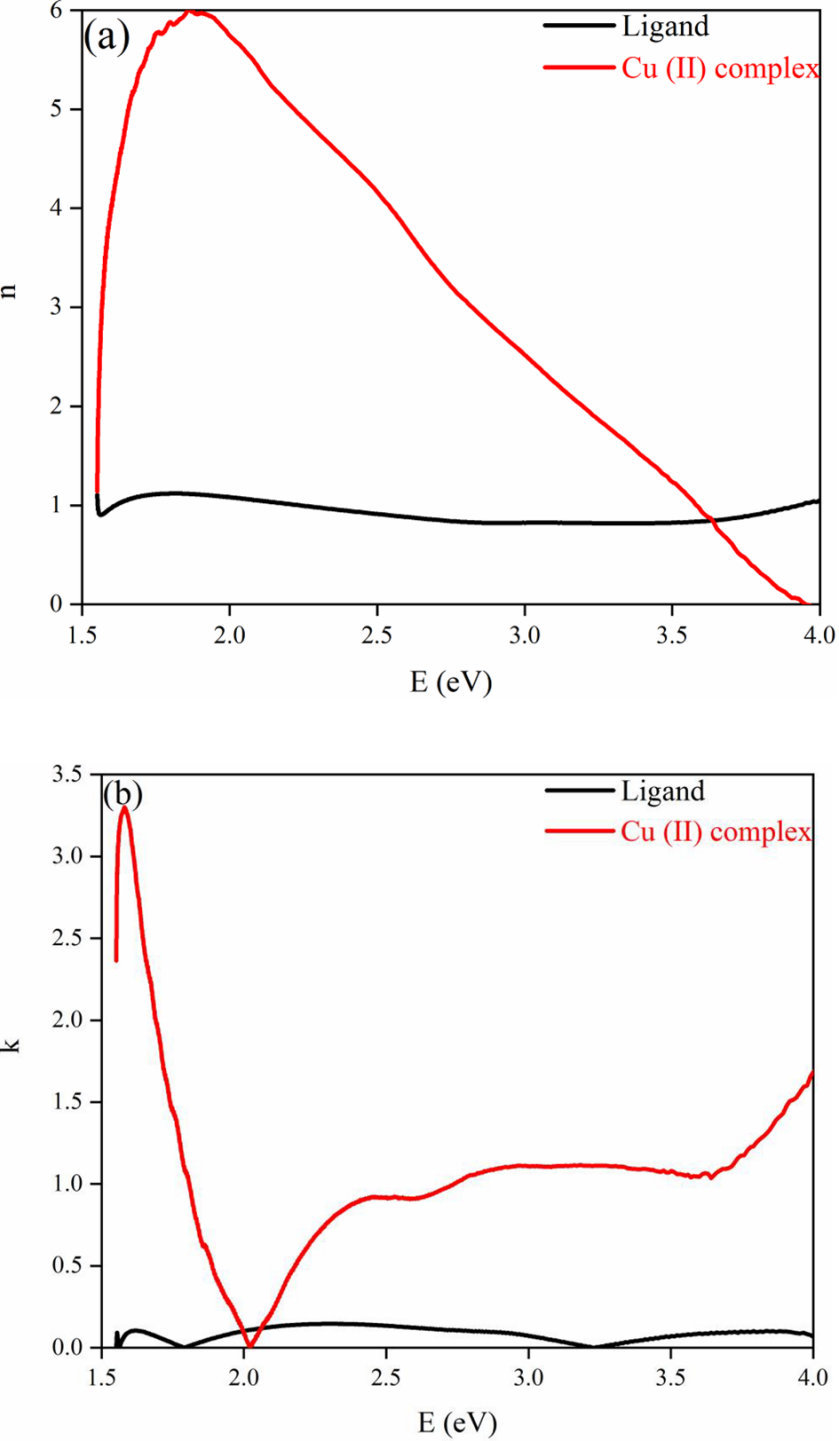
Refractive index (n) and extinction coefficient (k) for (**a**) Ligand; (**b**) Cu (II) Complex.

The values of n and k for the prepared samples at various wavelengths are depicted in Fig. 18a,b, respectively. The n values for Cu (II) Complex exhibit maximum values in the region of 1.8 eV < E < 2 eV, followed by a sharp increase in the region of 1.5 eV < E < 1.75 eV, which indicates a normal substantial dispersion. It has been observed that n values of the ligand are approximately constant but n values for Cu (II) Complex range widely from 1 to 6 with a decrement in wavelength from 2.25 eV < E < 4 eV. Additionally, a decrement in the n values originates from the preliminary band gap absorption. The lower n values have potential applications in optical devices such as n modifications in desired conductivity and mobility^42^. Moreover, it has been observed that the k-values of the ligand indicate approximately constant same as its n values, but for Cu (II) Complex represents a sharp decrement and reaches zero in, allowing the incident light to pass with negligible loss.

#### Nonlinear optical properties

**Figure 19:**
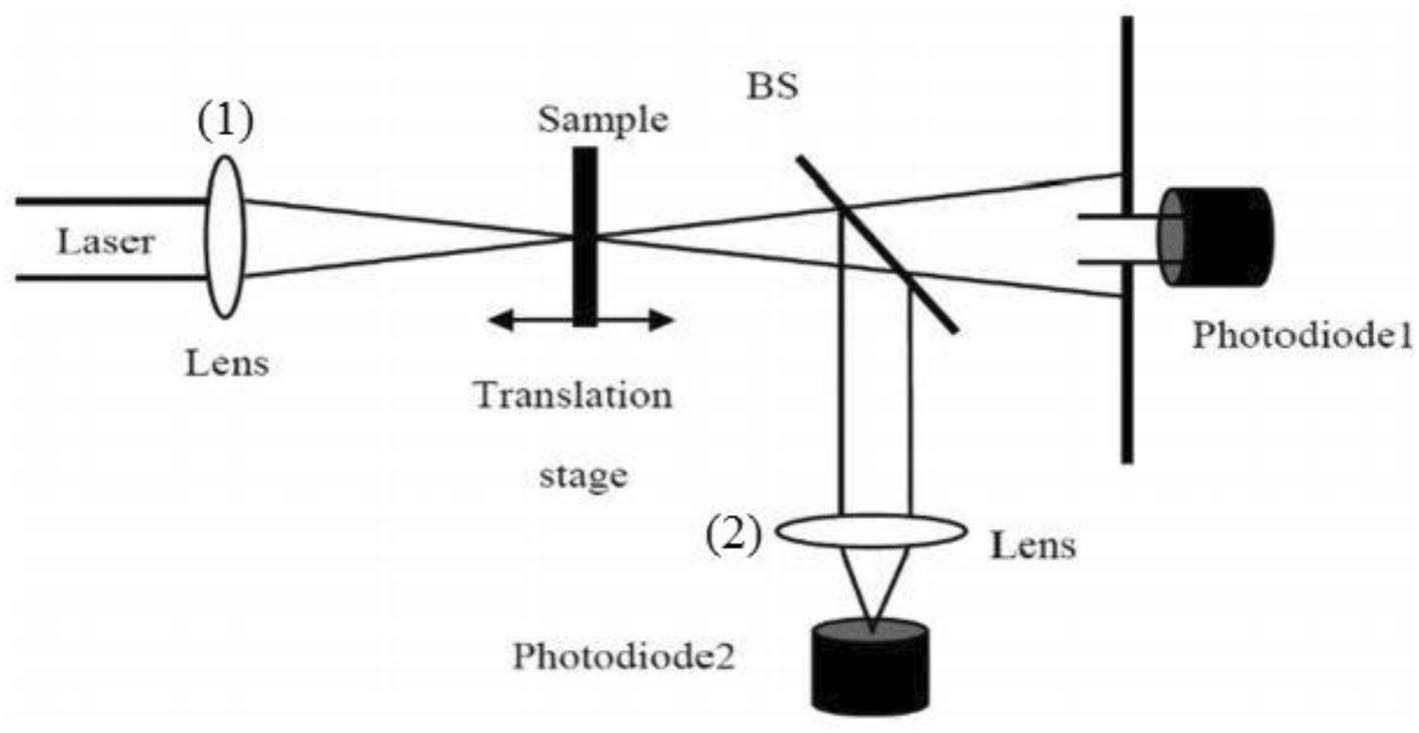
Scheme of the experimental setup of the Z-scan technique.

The Z-Scan method is used to investigate nonlinear refraction and absorption coefficients in material^77^. In this method, the nonlinear absorption coefficient ($\beta$) and the nonlinear refractive index ($n_{2}$) were evaluated through two closed (CA) and open (OA) aperture structures, respectively. With the help of the two photodiodes, the intensity dependant absorption and the fraction of diffracted intensity were measured in the OA and CA, respectively. The Z-scan is carried out through an Nd: YAG laser (532 nm). The laser incident power ($P_{0}$) into the synthesized samples was to be 29 mW, and the initial intensity of the laser beam $\left( {I_{0} } \right)$was 36.94 $\times$ 10^3^ $\left( {W/m^{2} } \right)$. The values of the $\beta$ and $n_{2}$ could be calculated via the recorded information on photodiodes 2 and 1, respectively. The schematic scheme of the Z-scan setup was represented in Fig. 19 where a Gaussian spatial profile and a continuous wave Nd–YAG laser was applied (wavelength = 532 nm). The beam splitter is named BS in Fig. 19 and the beam propagation is defined to be in the z-axis direction (from negative to positive z). To focus the laser beam, a positive lens at f = 10 cm was used. Photodiodes 2 and 1 measured the fraction of the diffracted intensity (in the CA technique) and the intensity dependant absorption (in the OA technique), respectively^78^.

In a nonlinear material, the intensity dependence of the refractive index, $$n$$, in a high-intensity laser beam can be expressed by $$n = n_{0} + n_{2} I$$ where $$n_{2}$$ and $$n_{0}$$ is called nonlinear and linear refractive index, respectively. This relation points out self-defocusing and self-focusing phenomena in the $$n_{2} < 0$$ and $$n_{2} > 0$$ cases, respectively. In the case of $$n_{2} > 0$$, the transmittance (T) of the photodiode (1) in Fig. 19 will show a valley and peak when the sample is scanned in the before and after of the focal point of the lens (1), respectively. In the case of $$n_{2} < 0$$, the valley and peak position in T in the before and after of the focal point of the lens (1) will be changed. On the other hand, the intensity dependence of the absorption coefficient of the sample in a high intensity could be exhibited by $$\alpha = \alpha_{0} + \beta I$$, where, $$\beta$$ and $$\alpha_{0}$$ is called nonlinear and linear absorption coefficient, respectively. When the sample is translated on stage, due to this relation, the information of photodiode (2) in Fig. 19 will represent a peak (because of nonlinear saturable absorption (SA)), and a valley (because of nonlinear two-photon absorption) in the $$\beta < 0$$ and $$\beta > 0$$$$>$$ 0, respectively.

**Figure 20:**
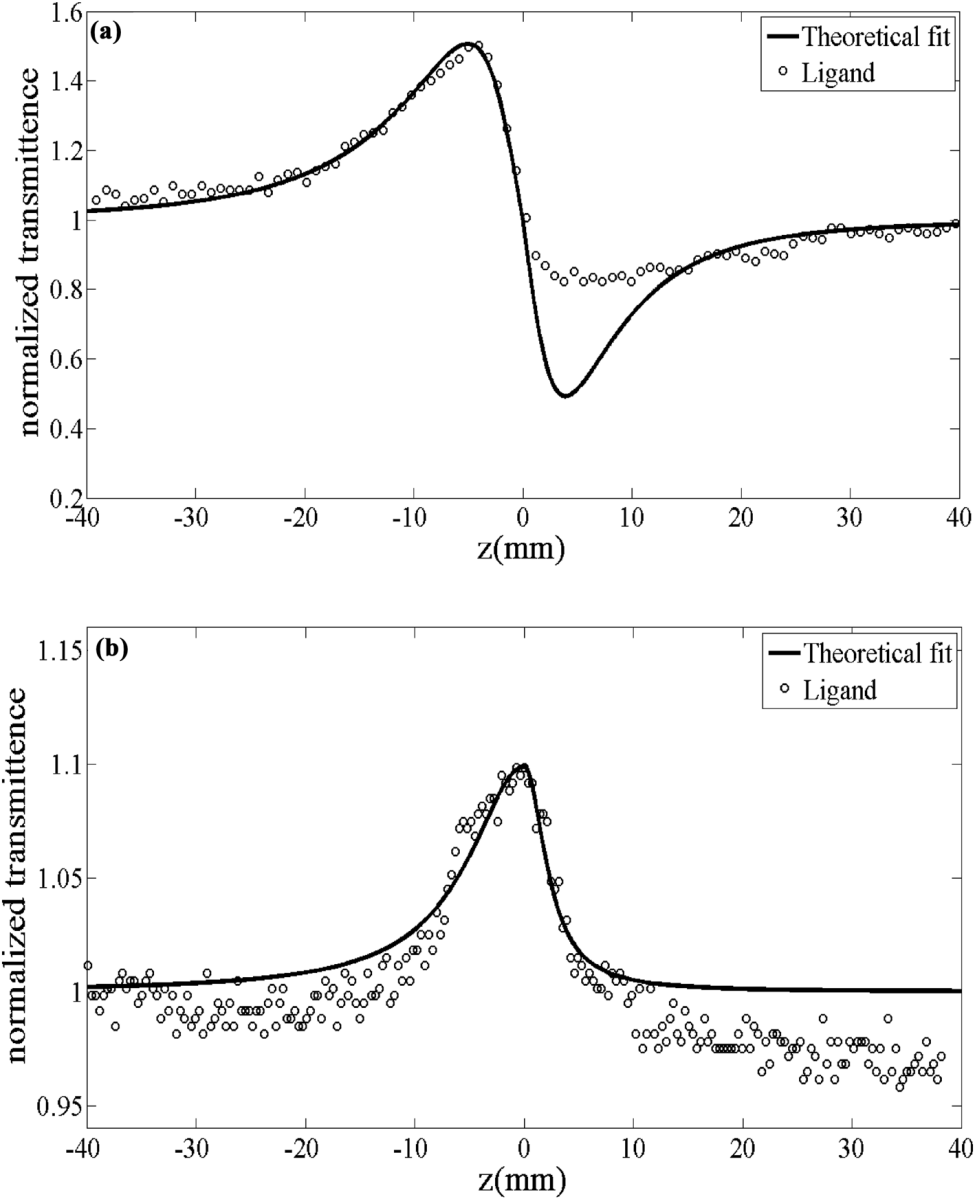
Z-scan plots of the ligand for (**a**) the closed, (**b**) the opened aperture.

**Figure 21:**
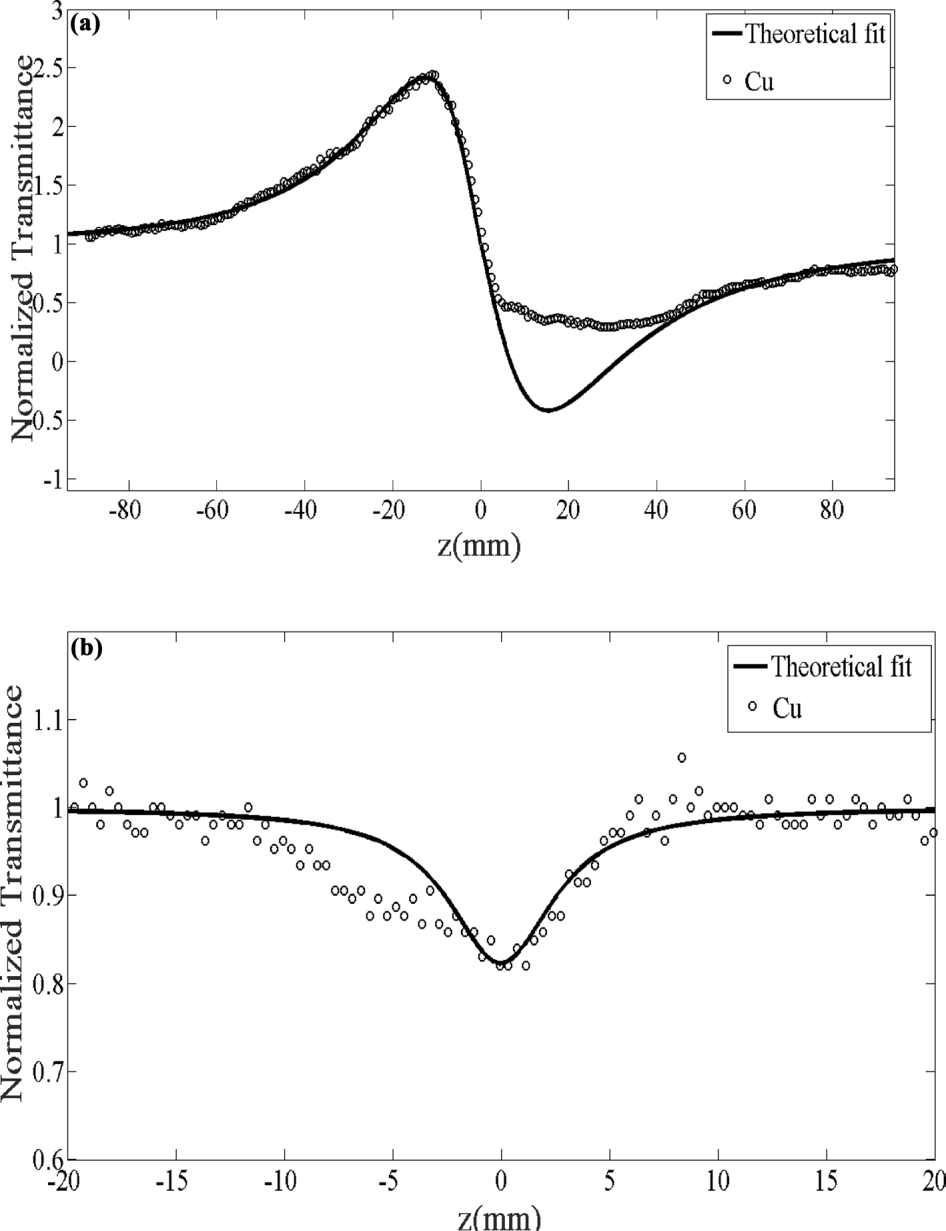
Z-scan plots of the Cu (II) Complex for (**a**) the closed, (**b**) the opened.

The CA and OA plots of the synthesized samples are exhibited in Figs. 20 and 21. As can be seen from Fig. 20a that expressed the CA Z-scan for the ligand, was initially a peak, and after that, a valley revealed transmittance, along with negative nonlinearity $\left( {n_{2} < 0} \right)$. The corresponding manner observed for Cu (II) Complex in Fig. 21a. In both CA Z-scan Figs. For ligand and Cu (II) Complex (Figs. 20a and 21a), the effect of the lensing is self-defocusing. On the other hand, Fig. 20b represents the OA Z-scan curve for the ligand where introducing a peak at z = 0 and hence exhibiting SA phenomena. The occurrence of the two-photon absorption is confirmed due to the existence of a valley in Fig. 21b for the Cu (II) Complex.

The $n_{2}$ can calculate from the following equation^79^:

8 $$ n_{2} = \frac{\lambda \Delta \phi }{{2\pi I_{0} L_{eff} }} $$

where the effective length is specified by:

9 $$ L_{eff} = \frac{{1 - e^{ - \alpha L} }}{\alpha } $$

where L is the sample length.

By fitting the experimental values obtained from CA Z-scan and combining them with the theoretical relation expressed in Eq (10), we can calculate the $\Delta \varphi$ amounts due to the intensity-dependent refractive index. Then n_2_ values could be estimated through Eq. (8)^78,79^.

10 $$ T(z) = 1 - \frac{{4\Delta \phi (z/z_{0} )^{2} }}{{\left( {1 + (z/z_{0} )^{2} )(9 + (z/z_{0} )^{2} } \right)}} $$

Here $z_{0} = k\omega_{0}^{2} /2$ called the Rayleigh diffraction length, $k = 2\pi /\lambda$($\lambda$ is the laser beam wavelength) and $\omega_{0}$ is the laser beam waist on the focal point of the lens (1).

In an OA evaluation, the $\beta$ values can be calculated through an excellent fitting relation theoretically and experimentally, which is plotted via Eq. (11) and computed via Eq. (12).

11 $$ \Delta T(z) \approx \frac{{q_{0} }}{2\sqrt 2 }\frac{1}{{\left( {1 + (z/z_{0} )^{2} } \right)}} $$

where

12 $$ q_{0} = \beta I_{0} L_{eff} $$

On the other hand, utilizing the Z-scan method, the third-order NLO characteristics of the ligand and its complex with Cu (II) were investigated. For this purpose, the magnitude of third-order nonlinear susceptibility $\left( {\chi^{(3)} } \right)$along with the imaginary and real portions of it were calculated with the help of the following relations^80^:

13 $$ {\text{Im}} \chi^{(3)} (esu) = \frac{{cn_{0}^{2} }}{{240 \, \pi^{2} \omega }}\beta_{eff} (m/w) $$

14 $$ {\text{Re}} \chi^{(3)} (esu) = \frac{{cn_{0}^{2} }}{{120 \, \pi^{2} }}n_{2} (m^{2} /w) $$

15 $$ \left| {\chi^{(3)} } \right| = \left[ {\left( {{\text{Re}} \chi^{(3)} } \right)^{2} + \left( {{\text{Im}} \chi^{(3)} } \right)^{2} } \right]^{1/2} (esu) $$

Obtained results and experimental details of the Z-scan analysis for the Cu (II) Complex and Ligand were summarized in Table 6. As can be concluded, compared to ligand, the Cu (II) Complex exhibited excellent and greater third-order NLO values. This response could be associated with a strong self-focusing effect. Besides, the negative n_2_ in an OA may attribute to the high (peak) intensity of transmittance at focus which demonstrated the existence of SA^46^. The positive $\beta$ value in the Cu (II) Complex represents the two-photon absorbance (TPA). The negative $\beta$ value in ligand could be associated with a lower dipole moment compared to Cu (II) Complex.

**Table 6:** Obtained results and experimental details of the Z-scan analysis for the Ligand and Cu (II) Complex. Nonlinear absorption coefficient $\left( \beta \right)$, Nonlinear refractive index $\left( {n_{2} } \right)$, Third-order nonlinear optical susceptibility $\left| {\chi^{(3)} } \right|$, The imaginary part of the third-order susceptibility $\left( {{\text{Im}} \;\chi^{(3)} } \right)$, The real part of the third-order susceptibility $\left( {{\text{Re}} \;\chi^{(3)} } \right)$, linear refractive index ($n_{0}$), linear absorption coefficient $\left( {\alpha_{0} } \right)$.

Variable/parameters	Values for compounds	
	Ligand	Cu (II) complex
$$n_{2}$$$$\times $$ 10^–10^ (m^2^/W)	−30.33	−88.69
$$\beta$$$$\times $$ 10^–5^ (m/W)	−410	748
${\text{Re}} \;\chi^{(3)} \times 10^{ - 3}$ (esu)	−1.58	−4.65
$\left( {{\text{Im}} \;\chi^{(3)} } \right) \times 10^{ - 3}$ (esu)	−0.30	0.55
$\left| {\chi ^{{(3)}} } \right| \times 10^{{ - 3}}$ (esu)	1.61	4.68
$\alpha_{0}$(1/cm)	0.56	1.02
$n_{0}$	1.436	1.438

## Conclusion

In the present work, *N*, *N′*-(1,2-diphenylethane-1,2-diylidene)bis(3-Nitrobenzohydrazide) and its Cu (II) complex were successfully synthesized and characterized. The obtained data from XRD and FT-IR verified their structure and composition. Also, in both synthesized Cu (II) complex and ligand samples, the XRD and FESEM calculations verified the nanocrystalline nature. The Rietveld technique was used with the *FullProf* software program Version 7.30. Magnetic investigation of the synthesized samples revealed the paramagnetic and weak ferromagnetism nature of the Cu (II) complex and the diamagnetic nature of the ligand. Evaluation of the DRS of the synthesized samples indicates higher reflectance amounts for Cu (II) complex compared to the ligand. Besides, in the absorbance spectra, two electronic transitions at 355 nm and 432 nm were observed for the ligand, which may originate from n → π^∗^ or π → π^∗^ transitions. With the help of the Kubelka–Munk theory and Tauc relation through reflectance data extracted from DRS, the band gap energies are estimated to be 2.67 eV and 2.89 eV for ligand and Cu (II) complex, respectively. The optical conductivity results were obtained to be 1.5 µSm^−1^, 13.7 µSm^−1^, and 102 µSm^−1^ for DMF, Ligand, and Cu (II) Complex, respectively. The calculation of both the refractive index and extinction coefficient, via the Kramers–Kronig studies, indicated maximum values at the same energy for the Cu (II) complex. The Z-scan data reveal that the both ligand and Cu (II) complex exhibit a strong self-defocusing effect. While, the ligand showed a negative and saturable, and Cu (II) complex exhibited a positive and two-photon nonlinear absorption, respectively. The magnitude of the third-order nonlinear susceptibility of the complex was examined to be 4.68 × 10^–3^ esu and 1.61 × 10^–3^ esu for Cu (II) complex and ligand, respectively. The obtained results of the Z-scan analysis revealed that the Cu (II) complex had improved the NLO characteristics of the ligand. A good synchronicity is observed between DFT-based calculations and experimental results. These results verified the potential usage of these materials (especially Cu (II) complex) in nonlinear photonic, nonlinear optical, and optoelectronic applications such as wavelength conversion and optical switching.

## Supplementary Information

Supplementary Information.

## Data Availability

All data generated or analyzed during this study are included in this published article.
