# Supplementary material for: Synthesis, characterization, and nonlinear optical properties of copper (II) ligand Schiff base complexes derived from 3-Nitrobenzohydrazide and benzyl
Source: Sci Rep. 2023 Jul 7;13:10988. doi: 10.1038/s41598-023-38086-w (PMC10328999; doi:10.1038/s41598-023-38086-w)
Supplement: Supplementary file 1 — Supplementary Information. [file 41598_2023_38086_MOESM1_ESM.docx]

| Coded Sample | R_F_ | R_Bragg_ | R_exp_ | R_wp_ | R_p_ |
| --- | --- | --- | --- | --- | --- |
| S_1_ | 4.82 | 6.17 | 4.78 | 42.7 | 27.3 |
| S_2_ | 5.01 | 8.29 | 5.43 | 104.0 | 55.5 |
| S_3_ | 1.55 | 2.50 | 4.77 | 30.4 | 19.4 |
| S_4_ | 1.25 | 1.83 | 3.96 | 15.3 | 11.4 |
| S_5_ | 4.08 | 6.43 | 3.96 | 16.2 | 11.6 |
| S_6_ | 12.2 | 12.6 | 3.95 | 11.18 | 8.81 |
| S_7_ | 12.9 | 14.7 | 3.95 | 14.8 | 10.4 |
| S_8_ | 3.25 | 1.70 | 3.94 | 11.0 | 8.0 |

Table *S_1_*. Crystallographic data of the used raw materials and the obtained complex product mixture.

By comparing the XRD data shown in figure *S_1_* for S_1_-S_3_, the data show that the used ligand is a mixture of monoclinic crystal system with space group of P12_1_/c1 and two kinds of cell parameters amounts including a:14.768, b:8.411, c:19.4802 Å, and β:123.338° for S_1_ (Reference code: 96-221-4146), and a:19.6970, b:7.152, c:12.949 Å, and β:100.34° for S_2_ (Reference doe: 96-201-3814). The Rietveld data show that the parameters R_F_, R_Bragg_, R_exp_, R_wp_, and R_p_ for S_3_ are lowest compared to S_1_ and S_2_. However, the proportion of S_1_ in the mixture is 91%. Also, by comparing the XRD patterns associated with Rietveld analysis data (figure *S_2_*) for the complex compound (S_4_-S_8_), we tested the synthesized complex by possible composite mixtures. At first, we analyzed the data when the CuSO_4_ crystal data (Space group: Pnma, a:4.85, b:6.62, c:8.38 Å, and α=β=γ=90°) were removed from the program and the data were achieved (S_4_ and S_5_). Then, the data were analyzed when only the ligands were used individually (S_6_ and S_7_). Finally, we examined the crystallographic data of the synthesized complex product mixture when the ligand and CuSO_4_ coexist in the final product (S_8_) in which the complex crystallized in monoclinic crystal system with the space group of C12/c1 and cell parameters of a:12.76, b:14.859, c:10.637 Å, and β: 114.777 ° (Reference code: 96-222-4482). According to Rietveld parameters data, it was found that best refinement (according to R_F_, R_Bragg_, R_exp_, R_wp_, and R_p_) was obtained for S_8_. So we conclude that the complex is a mixture of 4 crystal phases present in the product mixture.


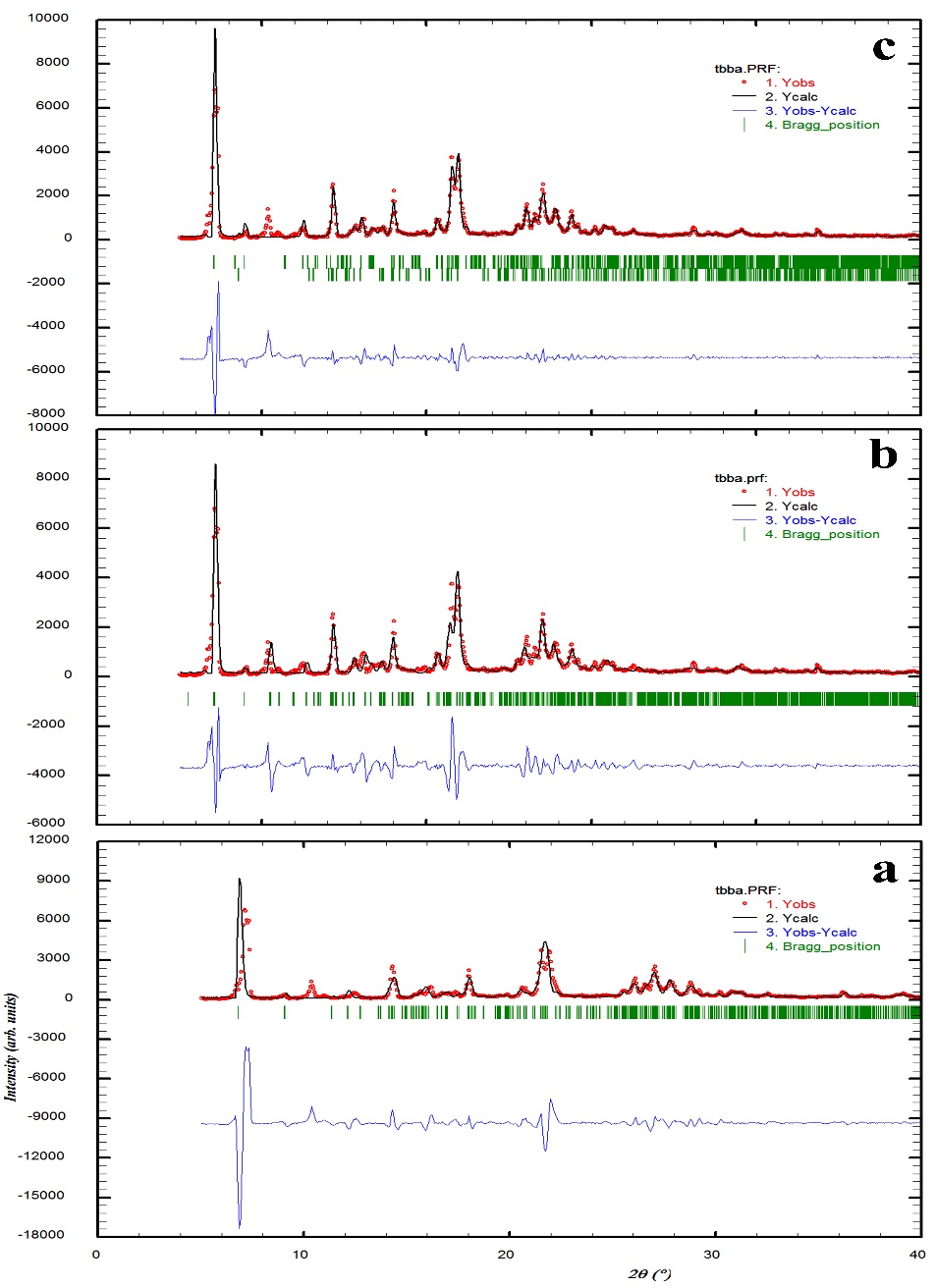


Figure *S_1_*. XRPD patterns associated with Rietveld analysis for a) S_1_, b) S_2_, and c) S_3_.


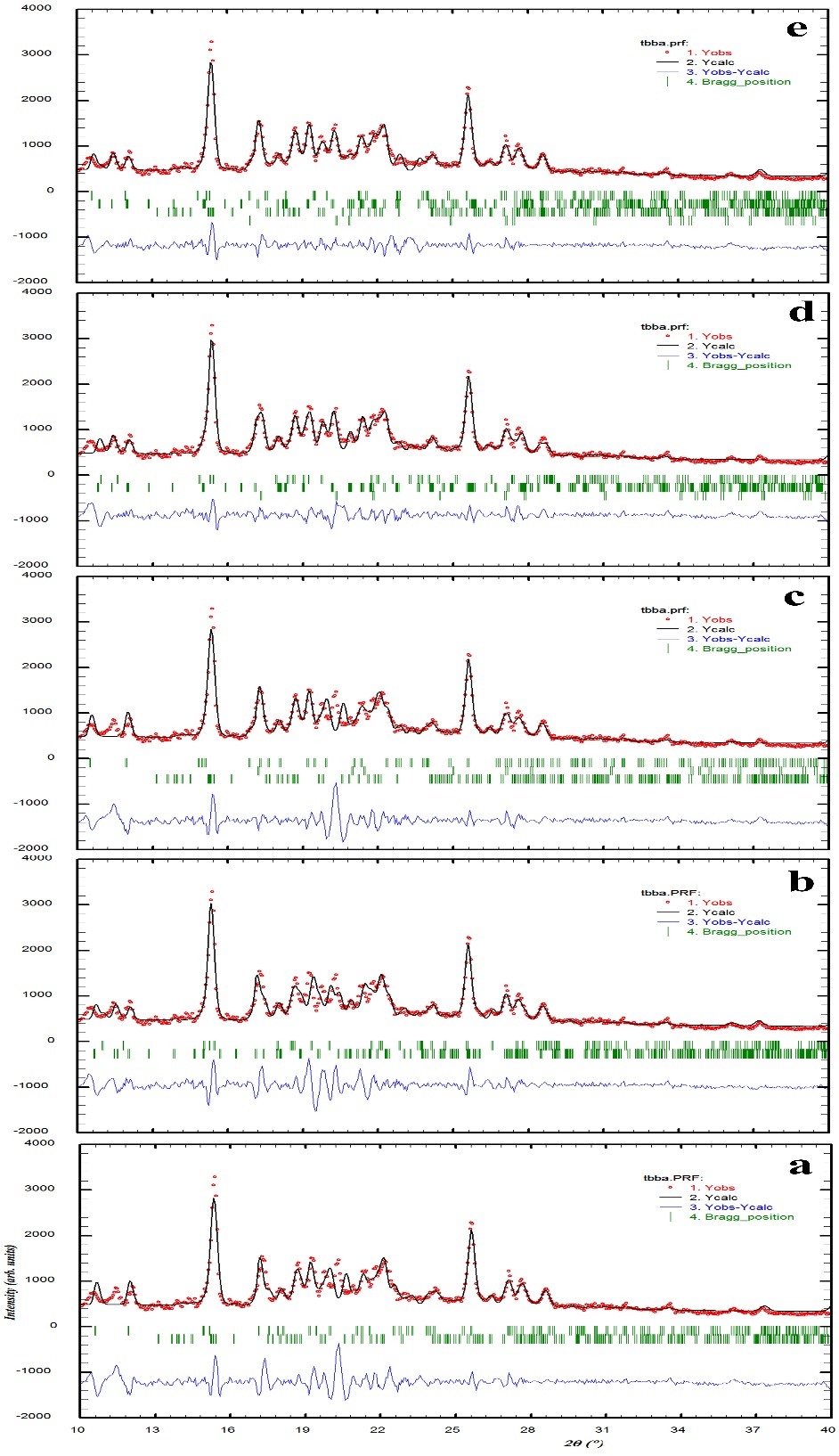


Figure *S_2_*. XRPD patterns of the complex product mixture where a) S_4_, b) S_5_, c) S_6_, d) S_7_, and e) S_8_.
